# Endothelium-Mimicking Multifunctional Coating Modified Cardiovascular Stents via a Stepwise Metal-Catechol-(Amine) Surface Engineering Strategy

**DOI:** 10.34133/2020/9203906

**Published:** 2020-04-24

**Authors:** Ying Yang, Peng Gao, Juan Wang, Qiufen Tu, Long Bai, Kaiqin Xiong, Hua Qiu, Xin Zhao, Manfred F. Maitz, Huaiyu Wang, Xiangyang Li, Qiang Zhao, Yin Xiao, Nan Huang, Zhilu Yang

**Affiliations:** ^1^Key Laboratory of Advanced Technologies of Materials, Ministry of Education, School of Materials Science and Engineering, Southwest Jiaotong University, Chengdu 610031, China; ^2^Institute of Health and Biomedical Innovation, Queensland University of Technology, Brisbane 4059, Australia; ^3^Australia-China Centre for Tissue Engineering and Regenerative Medicine, Queensland University of Technology, Brisbane 4059, Australia; ^4^Department of Biomedical Engineering, The Hong Kong Polytechnic University, Hung Hom, Kowloon, Hong Kong, China; ^5^Max Bergmann Center of Biomaterials, Leibniz Institute of Polymer Research Dresden, Hohe Strasse 6, 01069 Dresden, Germany; ^6^Institute of Biomedicine and Biotechnology, Shenzhen Institutes of Advanced Technology, Chinese Academy of Sciences, Shenzhen 518055, China; ^7^State Key Laboratory of Medicinal Chemical Biology, Key Laboratory of Bioactive Materials, Ministry of Education, College of Life Sciences, Nankai University, Tianjin, China

## Abstract

Stenting is currently the major therapeutic treatment for cardiovascular diseases. However, the nonbiogenic metal stents are inclined to trigger a cascade of cellular and molecular events including inflammatory response, thrombogenic reactions, smooth muscle cell hyperproliferation accompanied by the delayed arterial healing, and poor reendothelialization, thus leading to restenosis along with late stent thrombosis. To address prevalence critical problems, we present an endothelium-mimicking coating capable of rapid regeneration of a competently functioning new endothelial layer on stents through a stepwise metal (copper)-catechol-(amine) (MCA) surface chemistry strategy, leading to combinatorial endothelium-like functions with glutathione peroxidase-like catalytic activity and surface heparinization. Apart from the stable nitric oxide (NO) generating rate at the physiological level (2.2 × 10^−10^ mol/cm^2^/min lasting for 60 days), this proposed strategy could also generate abundant amine groups for allowing a high heparin conjugation efficacy up to ∼1 *μ*g/cm^2^, which is considerably higher than most of the conventional heparinized surfaces. The resultant coating could create an ideal microenvironment for bringing in enhanced anti-thrombogenicity, anti-inflammation, anti-proliferation of smooth muscle cells, re-endothelialization by regulating relevant gene expressions, hence preventing restenosis in vivo. We envision that the stepwise MCA coating strategy would facilitate the surface endothelium-mimicking engineering of vascular stents and be therefore helpful in the clinic to reduce complications associated with stenosis.

## 1. Introduction

Cardiovascular diseases (CVDs) are a group of medical conditions including heart and blood vessels flowing through the body. Every year, CVDs claim 17.9 million lives, representing 31% of global deaths [1]. Among the most common forms of CVDs, coronary heart disease happens when coronary arteries become narrow and hinder the blood flow to the heart [2]. Cardiovascular stent intervention in the clinic has benefited many patients with CVDs worldwide owing to the high efficiency of keeping the vessel open [3]. Nevertheless, in-stent restenosis (ISR) is still a major concern resulting from a cascade of molecular and cellular events associated with stenting, such as thrombus, inflammation, leukocyte accumulation, and hyperproliferation of smooth muscle cells (SMCs) [4]. Over the past two decades, numerous approaches (e.g., new drugs [5], polymer coatings [6], endothelial progenitor cell (EPC) capture [7], gene [8], and biomolecule modification [9, 10]) have emerged to ameliorate clinical outcomes. Nonetheless, substantial progress is limited such as the inability of using these strategies to facilitate the formation of a robust, competently functioning new endothelial layer. Since a healthy endothelium features the best property for preventing late stent thrombosis (LST) as well as in-stent restenosis [4], reconstruction/mimicry of endothelial cell function on the vascular stents would be the most rational way to improve vascular healing and prevent ISR after stenting.

**Figure 1:**
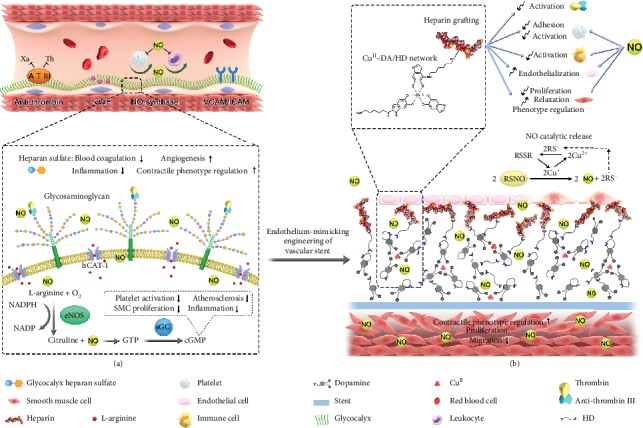
Scheme of metal-catechol-amine network- (MCAN-) based coating strategy for engineering endothelium-mimicking cardiovascular stent coating. (a) Functions of healthy ECs. HS on ECs has anticoagulant function through binding with antithrombin III (ATIII) to inhibit thrombin molecules existing in a coagulation system. The surface HS can also increase eNOS activity and inhibit ROS generation by polymorph nuclear and mononuclear leucocytes. Arterial ECs synthesize the vasodilator NO via eNOS and help to keep an anti-inflammatory and anticoagulant microenvironment. NO diffuses across the platelet or SMC membrane to activate the heterodimeric sGC. Then, sGC leads to increased expression of cGMP, further helping on inhibition of platelet activation as well as SMC proliferation, hinders inflammation and thrombus formation, and prevents ISR. (b) The MCAN-based coating strategy inspired by native endothelium function. The delicate organization of metal ion-Cu^2+^ in the MCAN gives GPx-like activity to the modified surface while a large amount of surface primary amine groups allows sufficient covalent grafting of heparin.

Endothelium plays a vital role in a number of processes, like hemostasis, angiogenesis, vascular permeability, and inflammation [11–14]. Under physiological conditions, endothelium synthesizes nitric oxide (NO) *via* catalytic reaction assisted by endothelial nitric oxide synthase (eNOS) and helps to keep an anti-inflammatory and anticoagulant microenvironment [15]. Synthesized NO diffuses across the platelet or SMC membrane to activate soluble guanylyl cyclase (sGC). Then, sGC leads to augmentation of cyclic guanylate monophosphate (cGMP) that helps to maintain a healthy vascular microenvironment (e.g., inhibiting platelet activation and SMC proliferation, hindering inflammation and thrombus formation, and even preventing ISR under pathological conditions (Figure 1)) [16, 17]. Unfortunately, the stenting process inevitably damages ECs, accompanied by impaired eNOS activity and insufficient NO production [18]. Although numerous NO-releasing/generating coatings, mostly by introducing NO supplier or catalyzer (like *N*-diazeniumdiolate or ascorbic acid) [10, 19, 20], have been developed to restore EC functions, these strategies have potential issues including the indetermination on precise therapeutic dosage as well as short half-life of NO supplier [21, 22]. Moreover, the amount of released NO may be compromised when deactivated by reactive oxygen species (ROS) during the EC repopulation after stenting. Therefore, further studies with considerations of the complex vascular microenvironment and the multifunction of endothelium need to be explored urgently for the development of vascular stent.

According to the above consideration, we intend to shift more attention to the molecular synergistic effects on the multifunction of ECs. As it is known, glycocalyx heparin sulfate (HS) is an analog of heparin and the most prominent component on the EC surface. It can increase eNOS activity [23] and inhibit reactive ROS generation by polymorph nuclear and mononuclear leukocytes (Figure 1) [24]. Moreover, HS exhibits anticoagulant function through binding with antithrombin III to inhibit thrombin molecules existing in coagulation cascade. Thus, vascular stents with NO-generating and HS-like properties may possess synergistically enhanced EC functions in the complex vascular microenvironment. Previous studies have integrated HS with EC-derived NO catalyzer to modify cardiovascular stent surfaces to reduce thrombus, enhance reendothelialization, and inhibit SMC proliferation [21, 25–33]. However, these cardiovascular stent coatings still suffer from weaknesses including the following: (1) the amount of integrated heparin and their physiological functions are compromised due to the limited surface anchoring sites (insufficient surface amine functional groups) [21]; (2) the NO release dosage is difficult to control: NO release normally exhibits an initial burst release (causes potential cytotoxic and mutagenic issues), followed by a short-term release (i.e., 4 weeks) at physiologically relevant levels, limiting their long-term clinical success (treatment no less than 6 months, suggested by clinical guidelines) [31, 34]; (3) the coatings which display synergistic heparin and NO generation properties usually have a thickness of a hundred micrometers [30, 32, 33]. Such thick layer artificially reduces the lumen area of blood vessels; additionally, they are often associated with insufficient flexibility and mechanical properties as well as weak adhesive strength to the stent surface.

In this work, we report a stepwise metal-catechol-amine strategy to engineer endothelium-mimicking stent surfaces with controllable heparin anchoring and NO release properties. The heparin-grafted and NO-generating coating reflects the physiological functions of native endothelium (Figure 1). After a facile one-step molecule/ion coassembling procedure, amine-bearing hexamethylenediamine (HD), glutathione peroxidase- (GPx-) like Cu^II^, and adhesive catechol dopamine (DA) easily formed the coating (Cu^II^-DA/HD) on a vascular stent. DA is a small biomolecule that mimics the adhesive component, L-DOPA, self-polymerizes, and forms a nanometer-thin adhesive layer of polydopamine (PDA) on stent surfaces. The Cu^II^-DA/HD networks endow the modified stent with GPx-like activity to Cu^II^ and catalytically decompose the endogenous S-nitrosothiols (RSNO) from fresh blood into NO continuously. Furthermore, the abundant primary amine groups from HD allow the covalent grafting of heparin. The combination of surface-bound heparin and catalytic NO contributes to the biomimetic assemblies of the endothelium, which can address the challenges of in-stent restenosis.

## 2. Results

### 2.1. Amine-Rich Metal-Catechol-(Amine) Surface Coating for NO-Generation

We selected amine-bearing HD, GPx-like copper ions from CuCl_2_, and adhesive catechol DA as candidate precursors to prepare the Cu^II^-DA/HD coatings, aiming to catalytically generate NO and provide sufficient reactive amine groups for heparin grafting *via* carbodiimide chemistry.

**Figure 2:**
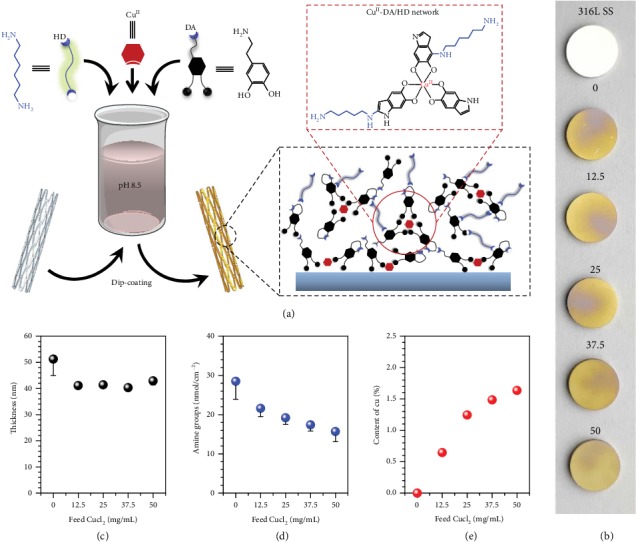
Amine-rich metal-catechol-(amine) surface coating for NO generation. (a) Schematic illustration of the one-step assembly of MCAN-based coatings on a 316L SS cardiovascular stents. Phenolic compound (dopamine (DA) and HD) and Cu^2+^ are chosen for preparing NO-generating coatings. By mixing Cu^II^, DA, and HD, the cardiovascular stent is facilely coated with Cu^II^-DA/HD film. (b) Images of 316L SS and Cu^II^-DA/HD coatings modified 316L SS with increasing CuCl_2_ feeding concentrations. The concentrations of CuCl_2_ were set at 0, 12.5, 25, 37.5, and 50 *μ*g/mL, respectively. (c) The thickness and (d) surface amine group density of the Cu^II^-DA/HD coatings with increasing CuCl_2_ feeding concentrations. (e) The atomic content of Cu of the Cu^II^-DA/HD coatings with increasing CuCl_2_ feeding concentrations obtained from XPS results. Data shown as the mean ± SD (*n* = 4).

Since the physiological functions of NO and heparin are dose-dependent, the Cu^II^-DA/HD coatings were designed with controllable content of Cu and density of surface amine groups. The Cu^II^-DA/HD coatings with graded composition were formed by applying a series of CuCl_2_ feeding concentration ranges of 0 to 50 *μ*g/mL, 2.44 mg/mL of HD, and 1 mg/mL of DA (Figure 2(a)). Resulting from the material versatility derived from DA, 316L stainless steel (SS, one common material of stents) substrates and complex shaped 316L SS cardiovascular stents were easily coated with mechanical flexible Cu^II^-DA/HD films by a one-step dip-coating process (Figure 2(b), Figure S1-2, Supporting Information). The Cu^II^-DA/HD coatings showed a typical dark-brown color of polyphenols, independent of the Cu^II^ content of the initial solution. As a coating used for surface engineering of stents, it presented high adhesive force and good flexibility to comply with deformation during stent implantation. The deposition success of the surface coatings was also evidenced by the thickness of 40 to 50 nm (Figure 2(c)) and the presence of signals of Cu, O, N, and C specific to the sample precursors of CuCl_2_, DA, and HD due to the X-ray photoelectron spectroscopy (XPS) results (shown in Supplementary Figure 3 and Table 1), respectively. A chemistry colorimetric method and XPS results revealed that the quantity of surface amine groups (Figure 2(d)) along with the contents of Cu (Figure 2(e)) was proportional to CuCl_2_ feeding concentrations, suggesting the feasibility of our developed metal-catechol-amine network (MCAN) strategy to tailor surface functionality of blood-contacting devices.

To illustrate the formation mechanism of the Cu^II^-DA/HD coating, electron paramagnetic resonance (EPR) and matrix-assisted laser desorption ionization mass spectrometry (MALDI-MS) were carried out (Figure S3, Supporting Information). EPR analysis revealed the involvement of coordination reaction (between DA and Cu^II^) in forming a Cu^II^-DA network in the coating (Figure S2A, Supporting Information), with the signals showing up at 3490 − 3430 mT. The [M+H]^+^ ion peaks at 361, 475, and 830 *m*/*z* indicated the possible formation of quadridentate, while the peaks of 632, 734, 894, and 1200 *m*/*z* stood for sexadentate, 802, 994, and 1110 *m*/*z* represented the hybrid coordination complexes evidenced by MALDI-MS spectrum. Meanwhile, the peaks of 711 *m*/*z* confirmed the participation of Schiff's base reaction as well as Michael addition between DA and HD. These findings indicate that the formation of Cu^II^-DA/HD coating not only involves ion assembly among the Cu^II^, DA, and DA/HD cross-linked complexes but also includes the molecular assembly of DA and HD.

### 2.2. Surface Grafting of Heparin on Cu^II^-DA/HD Coatings and NO Catalytic Release

**Figure 3:**
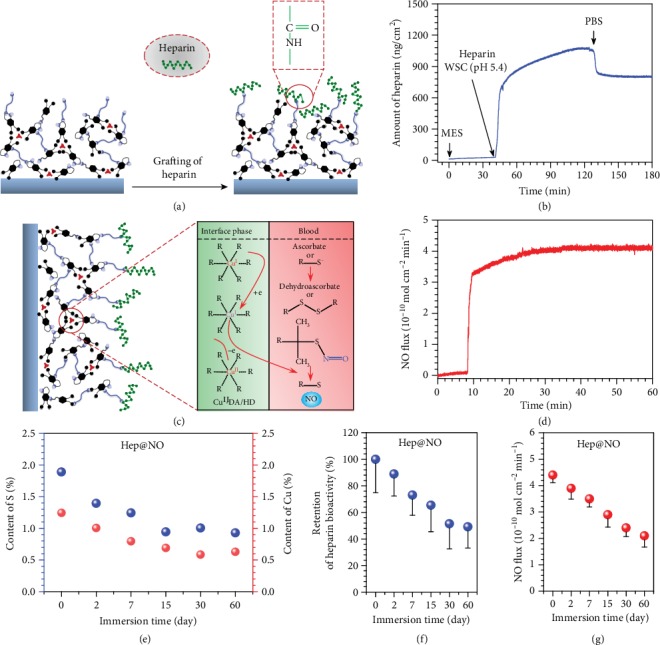
Surface grafting of heparin on the CuII-DA/HD coatings and NO catalytic release. (a) Schematic illustration (a) and QCM-D real-time monitoring (b) of heparin grafting onto the Cu^II^-DA/HD-coated surface using carbodiimide chemistry. (c) Schematic diagram of NO catalytic release mechanics. The Cu^II^ chelation endowed the coating with GPx-like property to decompose the GSNO that exists in the blood into NO at the interface. (d) NO catalytic production due to heparin-conjugated Cu^II^-DA/HD coating, determined in PBS (pH 7.4) supplied with 10 *μ*M GSH and 10 *μ*M GSNO at 37°C. (e) The S (left, blue) and Cu (right, red) signals resulting from Hep@NO-25 coatings after continuous exposure to GSNO solution (10 *μ*M in PBS (pH 7.4) with 10 *μ*M GSH) for up to 60 days. (f) Retained heparin activity and (g) NO catalytic generation after different periods of continuous Hep@NO samples exposure to donor solution. The heparin activity was determined using the anti-FXa assay. Data exhibited as the mean ± SD (*n* = 4).

Next, to develop an endothelium-mimicking biosurface, synergetic grafting of heparin with the primary amine groups existing on Cu^II^-DA/HD coating was performed through carbodiimide chemistry reaction (Figure 3(a)). In the following, the NO-generating coating of the Cu^II^-DA/HD is marked as NO, and the heparinized-Cu^II^-DA/HD is marked as Hep when without NO donor supplement, while as Hep@NO with NO donor supply (10 *μ*M GSNO, 10 *μ*M GSH).

Real-time quartz crystal microbalance with dissipation mode (QCM-D) was used to monitor heparin grafting onto the Cu^II^-DA/HD coatings. The grafted amounts ranged from 1064 to 732 ng/cm^2^ and were proportional to the density of surface amine groups (Figure 3(b), Figure S4, Supporting Information). Meanwhile, the emerging S2p signal of XPS spectra certified the conjugation success of heparin, most notably with the change trend of S contents in accordance with QCM results (Figure S5, Supporting Information). The bioactivity of the grafted heparin was detected through antifactor Xa (FXa) assay. It was found that the heparinized Cu^II^-DA/HD coatings with lower CuCl_2_ feeding (<37.5 *μ*g/mL) concentrations exhibited higher bioactivity (Supplementary Figure 6).

The incorporation of catalytic copper ions endowed the coating with GPx-like capability to catalyze NO release from RSNOs that exist in fresh blood (Figures 3(c) and 3(d)). As a transition metal-mediated catalysis, Cu^II^ is transferred to Cu^I^ by trace thiolate and further reacts with RSNOs to produce thiolate, NO, and Cu^II^ (Figure 3(c)). The NO production due to Cu^II^-DA/HD coatings before and after heparin grafting was detected by real-time chemiluminescence in PBS (pH 7.4) solution supplied with S-nitrosoglutathione (10 *μ*M, GSNO) and reducing agent glutathione (10 *μ*M, GSH) (Figure 3(d)). The NO release induced by the Cu^II^-DA/HD-coated surfaces before grafting of heparin exhibited dosage dependence on Cu^2+^ chelation to the Cu^II^-DA/HD network (Supplementary Figure 7A), demonstrating the controllability of MCAN surface chemistry in tailoring interface NO catalytic capacity. The conjugation of heparin did not compromise the catalytic NO formation property of the coating (Figure 3(d)). The heparinized-Cu^II^-DA/HD permitted facile tuning of the NO generation rate from 2.4 to 12.5 × 10^−10^ mol/cm^2^/min (Figure S7B, Supporting Information).

As a coating aiming for surface engineering of long-term serving biomedical implants, like cardiovascular stents, the stability of the NO production and the bioactivity of heparin are vital to guarantee the biological performance of heparinized Cu^II^-DA/HD *in vivo*. From this consideration, we further investigated the capacity of the heparinized Cu^II^-DA/HD to generate NO and maintain the bioactivity of heparin after continuously exposed in PBS with GSNO supply for up to 60 days. The changes in Cu and S contents of the coating, representing catalytic Cu and heparin, were determined by XPS, respectively. Although the contents of Cu and S reduced with the prolonged exposure time, Hep@NO showed high retention of S and Cu, where ~49% of the initial S and ~53% of the initial Cu were well maintained after 60 days (Figure 3(e)). Further evaluations of the catalytic NO release and anti-FXa activity revealed that the Hep@NO coating still exhibited a steady NO release flux of 2.2 × 10^−10^ mol/cm^2^/min (Figure 3(f)) and ~50% of the initial anti-FXa activity (Figure 3(g)). In conclusion, the durable NO generation and surface-anchored active heparin exhibited unique advantages for surface endothelium-mimicking engineering of blood-contacting devices without compromising either efficacy.

### 2.3. Regulation of the Growth Behaviors of Vascular and Immune Cells on the Endothelium-Mimicking Surface

The unavoidable injury to vessel wall injury due to stent implantation stimulates pathological responses, for example, thrombus formation, inflammation, and SMC migration, hence resulting in ISR. The formation of a dense and healthy EC layer plays a crucial role in preventing ISR. However, the proliferation of SMCs and inflammation are the two key factors for delayed reendothelialization. Therefore, it is crucial to endow vascular stents with selective regulation on the growth behaviors of the ECs, SMCs, and immune cell, in order to obtain successful reendothelialization.

As analyzed before, the physiological effects of heparin and NO are highly dose-dependent [35, 36]. Numerous studies have demonstrated that heparin was bound to a material surface in either a covalent or a noncovalent way showing anticoagulant properties and supporting EC growth along with an inhibitory effect on SMC proliferation if the grafted amount is located in a range of 300 to 3000 ng/cm^2^ [37]. An inhibitory effect on EC growth emerges if the immobilized heparin amount exceeds 3000 ng/cm^2 [^38^]^. In this work, our Cu^II^-DA/HD coatings provide the capacity to graft heparin ranging from 1064 to 732 ng/cm^2^ (Figure S4, Supporting Information), indicating the ideal dose of heparin for tailoring surface functionalities of vascular stent [39]. Besides, the highest NO flux resulting from Cu^II^-DA/HD coating reaches 12.5 × 10^−10^ mol/cm^2^/min (Figure S7B, Supporting Information), which would bring toxicity to vascular cells and tissue since the release rate exceeds the physiological NO flux (0.5-4 × 10^−10^ mol/cm^2^/min) [31]. However, tuning the NO release rate too low may also be insufficient for a therapeutic effect [40]. From this consideration, the impact of NO dose upon SMC and EC behaviors was investigated. We found that the increased release rate of NO, in the whole tested range, had increasing inhibitory effects on the SMC proliferation (Figure S8, Supporting Information). In the case of ECs, Cu^II^-DA/HD coatings with NO release above 6.6 × 10^−10^ mol/cm^2^/min did restrain human umbilical vein endothelial cell (HUVEC) growth (Supplementary Figure 9). This value was obtained for feeding concentrations of CuCl_2_ in the preparation of the NO-generating coatings exceeding 25 *μ*g/mL. Based on these results, the NO coatings of Cu^II^-DA/HD for further grafting heparin were all prepared with CuCl_2_ feeding concentration of 25 *μ*g/mL in following evaluations.

**Figure 4:**
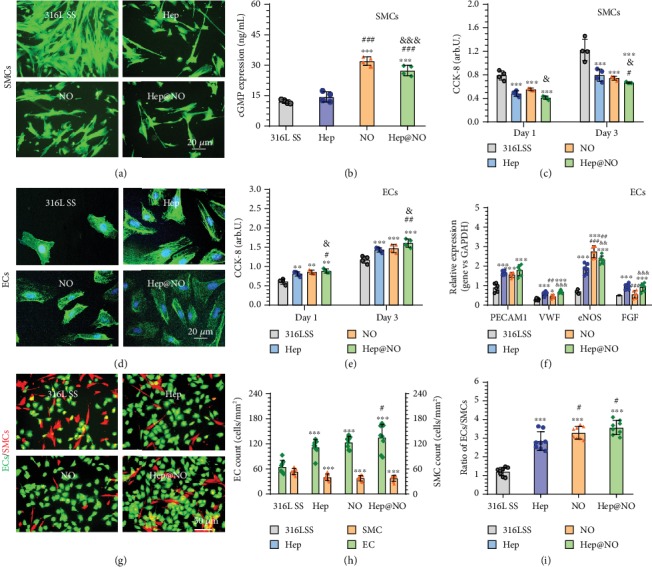
The growth behaviors of vascular cells on the endothelium-mimicking surface. (a) Fluorescence staining images of HUASMCs on bare 316L SS, Hep, NO, and Hep@NO coatings after 1 day of culture. (b) Corresponding concentration of cGMP in HUASMCs after 2 hours of culture. (c) Bioactivity of HUASMCs determined using CCK-8 assay after 1 and 3 days. (d) Fluorescence staining of HUVECs on the different surfaces (green: actin, blue: cell nuclei). (e) Proliferation of HUVECs after 1 and 3 days of culture determined using CCK-8 assay. (f) Gene expression of ECs after 3 days of culture (PECAM, VWF, eNOS, and FGF) determined by real-time quantitative polymerase chain reaction (RT-qPCR). Results were normalized by housekeeping gene GAPDH. (g) Co-culture of ECs/SMCs (HUVECs: green; HUASMCs: red) on the samples after 24 hours. (h) The counts of ECs (left, green) and SMCs (right, red) and (i) ratio of ECs/SMCs on samples surface. The data were collected from more than eight images. Data exhibited as the mean ± SD (*n* = 4, *n* = 6, or *n* = 8) and analyzed through a one-way ANOVA; ^∗^*p* < 0.05, ^∗∗^*p* < 0.01, and ^∗∗∗^*p* < 0.001 compared to 316L SS; ^#^*p* < 0.05, ^##^*p* < 0.01, and ^###^*p* < 0.001 compared to Hep; ^&^*p* < 0.05, ^&&^*p* < 0.01, and ^&&&^*p* < 0.001 compared to NO.

Fluorescence staining and vitality test with the metabolic Kit assay of human umbilical artery smooth muscle cells (HUASMCs) suggested that either Hep- or NO-functionalized surfaces significantly suppressed HUASMC growth compared to 316L SS control (Figures 4(a) and 4(c)). The dual Hep@NO functionalized surface remarkably restrained HUASMC growth compared with individually functionalized surfaces, exhibiting a synergetic effect of heparin and NO on SMCs suppression. The expression of cGMP in HUASMCs increased in the groups of NO and Hep@NO (Figure 4(b)), confirming that the inhibition of Cu^II^-DA/HD coatings on SMC proliferation was by specific cell regulation instead of toxicity.

For HUVECs, F-actin staining and CCK-8 tests unveiled that not only Hep- but also NO-coated surfaces supported cell spreading, development of cell cytoskeleton, and growth (Figures 4(d) and 4(e)), confirming the validity of the selected preparation conditions. Compared with the individually modified surfaces, the dual modified Hep@NO surface further promoted the HUVEC growth by forming a confluent monolayer with tightly arranged cells and very small intercellular space (Supplementary Figure 10). Gene expressions of platelet/EC adhesion molecule 1 (PECAM, CD31), eNOS, von Willebrand factor (vWF), and fibroblast growth factors (FGFs) were also detected. PECAM is specifically and constitutively expressed by all ECs that promotes EC migration and angiogenesis, functions as primary EC mechanosensor, and is responsible for stabilizing EC cell-cell junctions [41]. The eNOS has a protective function in the cardiovascular system, which is attributed to NO production [22]. While basal signaling of FGFs promotes junctional integrity in ECs via stabilization of VE-cadherin and catenins [42], Hep enhanced the expression of all four tested genes compared to the SS control. NO only enhanced PECAM and eNOS expression, and there was no obvious additive or synergistic effect of these stimuli (Figure 4(f)).

Comparing with the individual growth behavior of ECs and SMCs, the competitive behavior in coculture has been demonstrated to be more informative about the regeneration of an endothelial layer *in vivo* [43]. Such competitive attachment and proliferation behaviors of ECs and SMCs were investigated *via* seeding prelabelled HUVECs and HUASMCs with the ratio of 1 : 1 for 2 and 24 hours. Photographs (Figure 4(g), Figure S11A, Supporting Information), individual cell count (Figure 4(h), Figure S11B, Supporting Information) and number ratio of HUVECs to HUASMCs (Figure 4(i), Figure S11C, Supporting Information) demonstrated that the Hep and NO coatings endowed the functionalized surfaces with high EC selectivity. After 2 h, the EC adherent amounts on Hep and NO coatings increased by 42.5% and 38.4% compared to 316L SS, respectively. The dual functionalized Hep@NO surface promoted EC adhesion by 77.6%, while the adherent ratio of HUVECs to HUASMCs increased from 1.11 ± 0.10 (316L SS) to 2.49 ± 0.23. After 24 hours, the Hep- and NO-coated surfaces resulted in increased ratios of HUVECs to HUASMCs from 1.28 ± 0.31 (316L SS surface) to 2.9 ± 0.47 and 3.3 ± 0.31, respectively. The Hep@NO surface further promoted the competitive advantage of ECs over SMCs, as evidenced by the highest ratio of HUVECs to HUASMCs (3.5 ± 0.49). Thus, the coculture results along with the isolated EC and SMC culture tests illustrated that the Hep@NO coating provides a favorable microenvironment that selectively supports EC growth and suppresses SMC proliferation.

Atherosclerosis is a chronic inflammatory disease that can become severe with thrombosis or plaque rupture [44]. Inflammation plays a central role in the occurrence and development of spontaneous atherosclerosis and mechanically induced vascular injury [45]. Macrophages, as a key component of the immune system, regulate the inflammation process by secreting a variety of cytokines (like interleukins and tumor necrosis factor-*α* (TNF-*α*)). The inflammatory genes TNF-*α* and interleukin-1*β* (IL-1*β*) are upregulated after vascular injury and induce SMC proliferation and migration to the neointima [45]. Macrophages can be switched from the proinflammatory M1 to the prohealing M2 phenotype, along with plentiful signaling mediator syntheses that affect downstream EC performance [46]. Especially, the M2 marker IL-10 plays an essential role in restricting immune and inflammatory response, including inhibition of the production and activity of various proinflammatory cytokines like IL-6, IL-12, IL-18, IL-1*β*, and TNF-*α* [46]. Sophisticated physicochemical surface modifications allow precise control of multiple cell fates evidenced by recent development in materiobiology [47]. Thus, the behavior of macrophages was evaluated to explore the immune microenvironment response to different surfaces.

**Figure 5:**
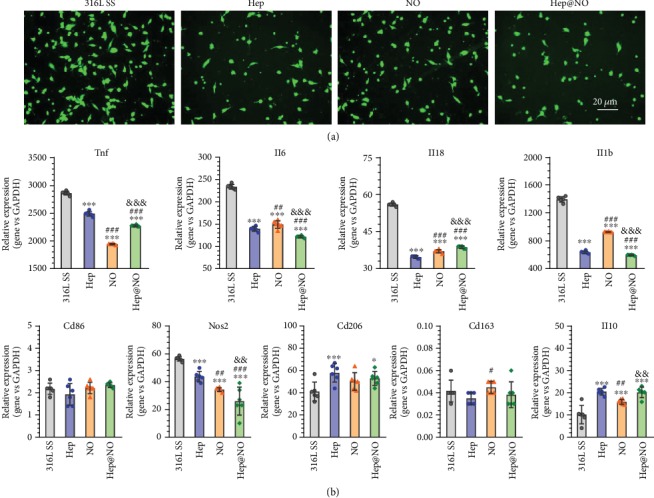
The growth behaviors of immune cells on the endothelium-mimicking surface. (a) Fluorescence staining images of macrophages on the 316L SS, Hep, NO, and Hep@NO samples. (b) Macrophage gene expression after culture on samples for 24 h and following stimulation with LPS for 6 h. Resulting influence of samples on inflammatory gene expression (TNF-*α*, IL-6, IL-18, and IL-1*β*), M1 (CD86 and iNOS) and M2 (CD206, CD163, and IL-10) markers were determined by RT-qPCR. Data exhibited as the mean ± SD (*n* = 6) and analyzed using a one-way ANOVA; ^∗^*p* < 0.05, ^∗∗^*p* < 0.01, and ^∗∗∗^*p* < 0.001 compared to 316L SS; ^#^*p* < 0.05, ^##^*p* < 0.01, and ^###^*p* < 0.001 compared to Hep; ^&^*p* < 0.05, ^&&^*p* < 0.01, and ^&&&^*p* < 0.001 compared to NO.

As shown in Figure 5(a), all four surfaces supported macrophage attachment, but notably more cells were adherent on bare 316L SS. A certain panel of cytokines and other markers was thereby analyzed to evaluate inflammation and macrophage differentiation. The introduction of heparin decreased macrophage adhesion and proinflammatory cytokine expression including inducible nitric oxide synthase (iNOS), TNF-*α*, IL-6, IL-18, and IL-1*β*. M2 markers mannose receptor cluster of differentiation 206 (CD 206) and IL-10 were significantly upregulated in the presence of heparin. NO has been reported to influence the functional polarization of macrophage towards anti-inflammatory M2 phenotype [48]. The results in Figure 5(b) demonstrated the downregulation of inflammatory genes (TNF-*α*, IL-6, IL-18, and IL-1*β*). Therefore, NO attenuates the inflammatory M1 polarization characterized by decreased iNOS secretion and increases the anti-inflammatory M2 polarization by increased IL-10 expression [48].

The optimized, dual-functional Hep@NO coating maintained the advantages of individual heparin and NO by downregulating the gene expression of the analyzed panel of inflammatory cytokines (Figure 5(b)). The M1 marker iNOS was inhibited due to coated surfaces, while M2 marker expression (CD206 and IL-10) was remarkably enhanced on Hep@NO coating. The pleiotropic influences were regulated through macrophage response to a variety of environmental signals that cause different functional phenotypes of proinflammatory M1 or anti-inflammatory M2 [48]. Herein, the synergetic modification of heparin anchoring and NO generation played an essential role in mediating M1 phenotype polarization to M2 phenotype, indicating a potential strategy to enhance tissue remodeling on vascular devices [49].

### 2.4. Anticoagulant Performances of the Endothelium-Mimicking Surface *In Vitro*

**Figure 6:**
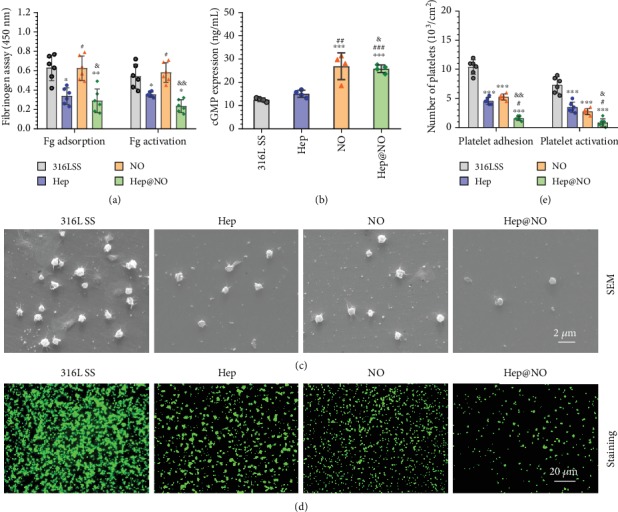
In vitro blood compatibility tests. (a) Relative quantify data of fibrinogen adsorption and *γ* chain exposure on 316L SS, Hep, NO, and Hep@NO samples. (b) The related cGMP expression in platelets after incubation of platelet-rich plasma (PRP) with samples for 30 min at 37°C, tested by ELISA kit. NO donor solution (10 *μ*M GSNO and 10 *μ*M GSH, PBS) was added to PRP. (c) Morphology of platelets on samples determined by scanning electron microscope (SEM). (d) Representative images of P-selectin staining, where activated platelets were marked by green fluorescence. (e) The numbers of the adherent and activated platelets on samples. Data exhibited as the mean ± SD (*n* = 4 or *n* = 6) and analyzed using a one-way ANOVA; ^∗^*p* < 0.05, ^∗∗^*p* < 0.01, and ^∗∗∗^*p* < 0.001 compared to 316L SS; ^#^*p* < 0.05 and ^###^*p* < 0.001 compared to Hep; ^&^*p* < 0.05 and ^&&^*p* < 0.01 compared to NO.

Foreign material in contact with blood leads to blood protein adsorption, complement system, and coagulation cascade activation, which further results in thrombus formation [50]. For blood-contacting implants, the thrombus formation is still a practical problem. The adsorption/activation of fibrinogen (Fg) and adhesion/activation of platelets are the two most fundamental contributors to thrombus formation [51]. As shown in Figure 6(a), both Hep and Hep@NO surfaces significantly decreased the adsorption and activation of Fg, determined by immunochemistry with universal and conformation-specific antibodies. However, the NO surface did not alter the Fg behavior compared to bare 316L SS (Figure 6(a)). Consequently, the combination of NO with heparin induced the same response of Fg as the Hep surface.

The mechanism of NO to suppress platelet adhesion/activation is mediated by inhibiting the thromboxane A_2_ receptor, which belongs to cGMP-dependent protein kinases [52]. To confirm the physiological effect of NO, the expression of cGMP in platelets after 2 hours of culture with the uncoated and coated surfaces was tested. The two NO and Hep@NO coatings both induced increased cGMP synthesis (Figure 6(b)). Additionally, the cGMP expression induced by the NO surfaces was correlated with the NO dosage (Supplementary Figure 12A). There were severe platelet aggregation and activation on 316L SS, with the highest amount of adhered platelets on all surfaces (Figures 6(c), 6(d), and 6(e)). A limited number of platelets with lower activation levels were exhibited on Hep surface. NO surface also remarkably inhibited adhesion and activation of platelets, and the inhibitory effect graded with the feed of CuCl_2_ used for fabricating the Cu^II^-DA/HD (Figure S12B, Supporting Information). This highlights the impact of the NO-cGMP pathway on platelet function, as well as the dose dependence of platelet function on NO concentrations. The combination of Hep with NO showed synergetic effects on platelets, with an impressively enhanced suppression on platelet attachment and activation. For the Hep@NO surfaces, only a small number of platelets existed; the majority were round, suggesting a nonactivated, resting state. The results suggest that the synergetic modification by NO and heparin reduced Fg adsorption and activation and especially kept platelets in a quiescent state, hence promising an enhanced reduction in thrombogenicity *in vitro*.

### 2.5. *Ex Vivo* and *In Vivo* Hemocompatibility of the Endothelium-Mimicking Surfaces

**Figure 7:**
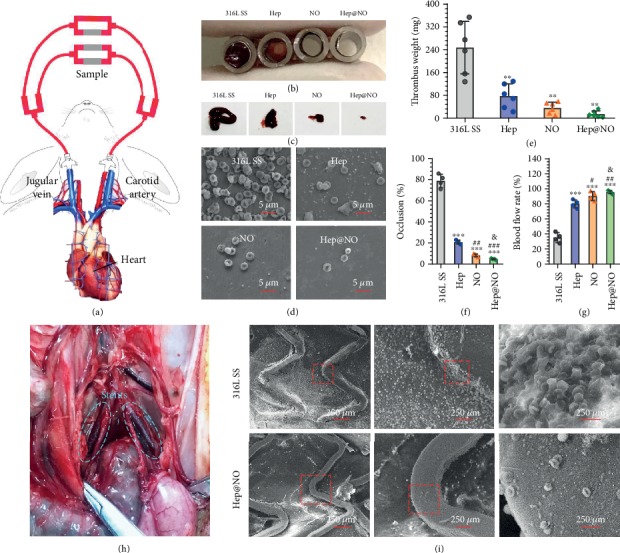
Ex vivo and in vivo hemocompatibility of the endothelium-mimicking surfaces. (a) Ex-vivo circulation thrombogenicity assay of Hep, NO, and Hep@NO samples determined by an arteriovenous shunt model with the supplement of NO donor. (b) Cross-sectional tube images after 2 hours of blood flow without additional heparin. (c) Thrombus formation on 316 L SS samples before and after Hep, NO and Hep@NO functionalization. (d) SEM, (e) thrombus weight, (f) occlusion rates of circuits, and (g) rate of blood flow by the end of the circulation experiments due to different treatments. (h) The bare 316L SS cardiovascular stents and Hep@NO-coated stents were placed at the iliac artery under angiographic control. The stents were harvested after 2 hours to check the hemocompatibility. (i) SEM results of the harvested stent. Data exhibited as the mean ± SD (*n* = 4 or *n* = 6) and analyzed using a one-way ANOVA; ^∗^*p* < 0.05, ^∗∗^*p* < 0.01, and ^∗∗∗^*p* < 0.001 compared to 316L SS; ^#^*p* < 0.05, ^##^*p* < 0.01, and ^###^*p* < 0.001 compared to Hep; ^&^*p* < 0.05, ^&&^*p* < 0.01, and ^&&&^*p* < 0.001 compared to NO.

To further check the synergetic effects of heparin and NO in reducing thrombogenicity, we performed *ex vivo* and *in vivo* antithrombogenic evaluations. An arteriovenous shunt model was applied for the *ex vivo* antithrombogenic test, as reported before [51]. The custom-built extracorporeal circuit (ECC) was set up by connecting left carotid artery and right external jugular to guarantee blood flow, with samples rolled and installed in the middle part of ECC (Figure 7(a)). After 2 hours of flow circulation, the circuits of Hep, NO, and Hep@NO coatings presented remarkable reductions in occlusive thrombosis (Figure 7(b) and 7(c)). SEM images of the uncoated 316L SS sample showed severe thrombus consisted of activated platelets, red blood cells, and fibrin networks, whereas the coated samples significantly reduced the platelet activation, formation of fibrin networks, and inclusion of red blood cells (Figure 7(d)). There were still a few activated platelets and fibrin shown on the Hep- or NO-coated surfaces, whereas there are a minimal number of red blood cells without any activated platelets and fibrin on dual active Hep@NO-coated surface. The total weight of the thrombi formed on the Hep@NO-coated surfaces was decreased by 18-fold, 5.1-fold, and 1.6-fold as compared with 316L SS, Hep, and NO, respectively (Figure 7(e)). The occlusion rates, calculated based on lumen areas of the circuits containing the tested samples, showed severe occlusion for the uncoated 316L SS (79 ± 5% decrease in the lumen area), whereas the Hep- and NO-coated groups showed occlusion rates of 20 ± 1.4 and 8 ± 1.1%, respectively. The dual functionalized Hep@NO-coated group showed an occlusion rate of 4 ± 0.6% (Figure 7(f)). As a result, the Hep@NO group exhibited the highest blood flow rate of 94 ± 4% after 2 hours of circulation (Figure 7(g)). In contrast, the circuit with the uncoated 316L SS retained only a 35 ± 5% blood flow rate. All the *ex vivo* data proved that the synergetic modification strategy through the combination of surface heparin anchoring and interfacial NO catalytic release enabled efficient endothelium-mimicking engineering of vascular devices with excellent antithrombogenic properties.

To further confirm the feasibility of our Hep@NO coatings as an antithrombotic coating for vascular devices, uncoated and Hep@NO-coated 316L SS stents were parallelly placed into iliac arteries of New Zealand white rabbits (Figure 7(h)). After 2 hours, the blood vessels with stent were harvested to assess acute thrombus formation. SEM (Figure 7(i)) images of the uncoated 316L SS stent showed thrombi consisting of dense fibrillar networks of polymerized fibrin-bound activated platelets and red blood cells, whereas only a few red blood cells and round platelets are present on Hep@NO-coated stents.

### 2.6. *In Vivo* Therapeutic Effects of Endothelium-Mimicking Stents

**Figure 8:**
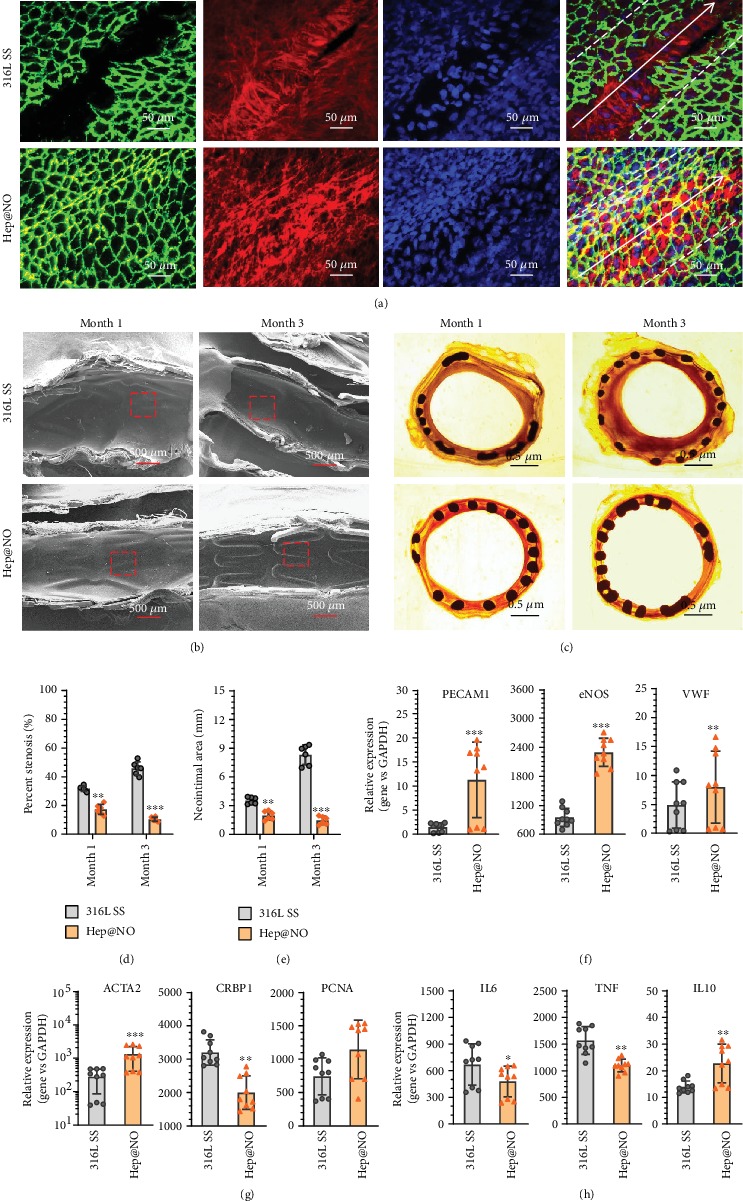
Stent implantation in vivo. (a) CLSM images (green: CD31, red: F-actin, blue: cell nucleus) reveal the endothelialization on the stents (outlined by the dashed lines). (b) The morphology of Hep@NO-coated and bare stents after implantation for 1 and 3 months, evaluated by SEM. (c) Influence of implanted stents on ISR, determined through histomorphometric analysis. (d) Percentage of stenosis and (e) mean neointimal area analysis indicated significant reduction of ISR by the Hep@NO coating. Gene expression of the harvested tissue with stent detected by RT-qPCR. (f) EC-related genes: PECAM, eNOS, and VWF; (g) SMC-related genes: *α*-SMA, cRBP-1, and proliferating cell nuclear antigen (PCNA); (h) gene expression levels of inflammatory cytokines IL-6 and TNF-*α* and M2 marker IL-10. Data presented as the mean ± SD (*n* = 6 or *n* = 9) and analyzed using a one-way ANOVA; ^∗^*p* < 0.05, ^∗∗^*p* < 0.01, and ^∗∗∗^*p* < 0.001.

To further test the ability of Hep@NO coating for tailoring surface endothelium-mimicking functionality of vascular stents on reendothelialization and restenosis, the long-term rabbit stent implantation tests were performed. The capacity of rapid reendothelialization on vascular stents determines its long-service outcome; the immunofluorescence staining and identification of the newly formed tissue were therefore performed. As the CLSM images shown in Figure 8(a) and Figure S13, Supporting Information, there were sparse and discontinuous ECs on the bare 316L SS stent strut after one week's implantation, suggestive of either SMC overproliferation or inflammation in the unendothelialized area. Meanwhile, the Hep@NO-coated stent strut was fully covered by a complete new endothelial layer, providing a better microenvironment for EC migration from the surrounding tissue and proliferation. It is clear that the endothelialization level of Hep@NO-coated stent struts was higher than 316L SS control stent. The SEM images of the stented iliac arteries revealed that there were no cells with endothelial morphology, but fiber-like tissues grown on the bare 316L SS stents after implantation for 1 month (Figure 8(b), Figure S14A, Supporting Information). In the case of 3 months, although most of the bare 316L SS stent struts were covered by cells, the cells were nonisotropic and did not grow along with the direction of blood flow. The impure EC and restenotic tissue further led to intimal hyperplasia. In contrast, a dense endothelial layer thoroughly covered the Hep@NO-coated stents after 1 month, grown along the direction of blood flow, indicating that the Hep@NO coating provided a favorable microenvironment for the reendothelialization process. The stent profile was still visible in the group of Hep@NO-coated stents after 3 months. To examine the effect of stents on restraining ISR and intimal hyperplasia, histomorphometric assay was thereby performed (Figure 8(c), Supplementary Figure 14B). The Van Gieson's staining exhibited a conspicuous suppression on neointimal stenosis (Figure 8(d); 16.2% ± 5.3%*vs.*31.8% ± 2.2% after 1 month, 10.8% ± 2.6%*vs.*46.0% ± 7.7% after 3 months) and mean neointimal area (Figure 8(e); 2 ± 0.4 mm^2^*vs.*3.5 ± 0.31 mm^2^ for 1 month, 1.4 ± 0.37 mm^2^*vs.*8.0 ± 1.0 mm^2^ for 3 months) for the Hep@NO-coated and control stents, respectively.

To thoroughly understand the *in vivo* effects of the Hep@NO coating on ECs, SMCs, and immune cells, gene expression of *in situ* stented arteries was performed. RT-qPCR analysis revealed that the Hep@NO-coated stents impressively upregulated the expression of PECAM, eNOS, and vWF that are individually and inducibly expressed by endothelium (Figure 8(f)), confirming that the Hep@NO coating facilitated the regeneration of a healthy endothelial layer *in vivo*. Meanwhile, the ability of a stent to regulate the proliferation and phenotype of SMCs is also crucial to prevent ISR complication. [43] As shown in Figure 8(g), Hep@NO-coated stents induced the contractile phenotype gene expression of *α*-smooth muscle actin (*α*-SMA) and suppressed the SMC dedifferentiation marker of cellular retinol binding protein 1 (cRBP-1). This indicates that the Hep@NO functionalized stents significantly resisted intimal hyperplasia by synergistically inhibiting the excessive proliferation of SMCs and regulating their phenotype to contractile type. Inconsistent with the *in vitro* results of macrophages, the Hep@NO-coated stents remarkably downregulated the gene expression of the inflammatory cytokines IL-6 and TNF-*α*, meanwhile upregulating M2 marker expression of IL-10 (Figure 8(h)). This illustrates that the combination of heparin with NO endowed the modified stents with the ability to synergistically mediate M1 phenotype polarization to M2 phenotype, hence resulting in the attribution to the inhibitory effects on intimal hyperplasia *in vivo*.

## 3. Discussion

The nature shows uncanny workmanship and delicate materials/structures and offers spirited inspiration to design biomimic materials given the variety of elaborated properties [53, 54]. For the lumen of blood vessels, the endothelium serves as a structural barrier between the blood and vessel walls with functions of vasodilation, oxidative protection, anti-inflammation, thrombolysis, and antiproliferation [15, 17, 55, 56]. The endothelial function therefore becomes an important physiological target for new therapy and facilitates options to improve current agents. As is known, the functionality of healthy endothelium is much dependent on the dynamic balancing production of tissue factor, cytokines, peptides, and signal molecules responding to various stimuli [15]. By incorporating functional peptides, growth factors, antibodies to mimic EC extracellular matrix, rapid endothelialization, and improved endothelial function were achieved [57–59]. Of great importance is the fact that NO is an essential signaling molecule transiently produced by endothelium and diffuses throughout the lumen and vessel wall, leading to angiogenesis and SMC relaxation, inhibition of platelet activation, and aggregation and further thrombosis, as well as regulation of immune cells fate [22, 26]. The endothelial glycocalyx, a slippery smooth gel coating on the endothelium, is the actual interface with the circulating fluids [60, 61]. Among the proteoglycans, heparan sulfate accounts for ~50-90% of the total amount existing in glycocalyx [62].

There were explorations in mimicking endothelium for blood-contacting devices, from single or multiple functions [12, 13, 27]. Meyerhoff's lab described a polymeric coating which combined NO production with heparin bounding to obtain the nonthrombogenic character of endothelium [33]. The heparin showed anticoagulant activity, and NO flux could be regulated from 0.5 to 60 × 10^−10^ mol/cm^2^/min for different periods of 24 h to 1 week. Furthermore, multifunctional bilayer coatings were endowed with not only controllable NO production but also active thrombomodulin (TM) and heparin conjugation. By changing the coating thickness, stable NO production at physiological levels was extended to 2 weeks [32]. Recently, several Cu(II)-ligand complexes were investigated to endow intravascular catheters with on demand NO generation for thromboresistant and antimicrobial performance [63, 64]. The efficiency and stability of NO generation were well compared by equipping catheters with different Cu(II)-ligand complexes through electrocatalytic nitrite reduction. The optimized complexes remarkably decreased microbial biofilm formation on catheter exposed to bacteria growth for over 5 days with NO flux (as low as 0.7 × 10^−10^ mol/cm^2^/min) on for 3 or 6 hours per day [64]. Kipper's lab has reported NO donor-loaded polysaccharides with the capacity of continuous NO production for ~80 h, resulting in suppression on platelet activation as well as antimicrobial property [65]. Later on, they reported a glycocalyx-inspired NO-releasing surface that exhibits remarkable inhibition on platelet and leukocyte adhesion/activation [30]. The combining glycocalyx-inspired features of nanostructured surfaces, polysaccharide-based multilayers, and NO-donor chemistry endowed biomaterials surface with multiple functions. In our recent study, the sequential conjugation of heparin and selenocystamine onto an amine-bearing film was also developed [21]. However, generating a new biomedical device coating with therapeutic delivery property should not only include the off-target effects and duration of delivery but also potential side effects and coating longevity. Thus, current strategies still could not fulfill the duration requirements for long-term blood-contacting devices and other essential functions. In our work, the long duration of NO release and heparin activity was due to the longevity and delicate organization of the coating. The coating demonstrated dose controls of NO catalytic release and heparin immobilization, especially the durability in their dose and retention of bioactivity. The bioactivity of anchored heparin was well maintained even after 60 days with high retention of ~50% anti-FXa activity and S content. Meanwhile, there were still ~53% of Cu contents maintaining with the capacities of NO release flux of 2.2 × 10^−10^ mol/cm^2^/min for the Hep@NO group.

Multiple lines of evidence confirm that inflammatory is highly involved in all phases of atherosclerosis [44]. It is worth noting that the stent implantation also causes mechanical injury which leads to local inflammatory, SMC proliferation, extracellular matrix secretion, and even neointimal thickening and restenosis [45]. As essential parts of the immune system, macrophages respond to foreign stent and provoke inflammation through secreting various cytokines and signaling mediators (like iNOS and VEGF) that affect downstream EC performance [46]. Heparin exerts immune-modulatory and anti-inflammatory actions, evidenced by the upregulated M2 markers CD206 and IL-10 and downregulated M1 marker iNOS expression [66]. In the presence of heparin, inflammatory cytokine expressions of TNF-*α*, IL-6, and IL1*β* were also reduced (Figure 8(h)). NO stimulates expression of collagenase matrix metalloproteinases-13 (MMP-13), which mediated the expression of iNOS and further promoted M1 macrophages transformation to M2 [67]. For the *in vivo* results, the Hep@NO-modified stents attenuated inflammatory and mediated macrophage polarization to M2 phenotype by downregulating inflammatory cytokines including TNF-*α* and IL-6 and upregulating M2 marker expression of IL-10. By applying inflammatory regulating Hep@NO coating, the lesion-healing challenges due to atherosclerosis and mechanical vascular injury could be improved. Under a prohealing microenvironment, the normal functions of endothelium could therefore be guaranteed to determine the fate of vascular stents.

Due to the short half-life of NO and *in situ* precise dosage requirement, the optimized physiological doses here are expected to exhibit no off-target effects or postpone endothelialization [68]. It is worth noting that the heparinized MCAN permitted precise dosage control of therapeutic NO production in the range from 2.4 to 12.5 × 10^−10^ mol/cm^2^/min by applying a series of initial Cu^II^ feeding concentrations (Figure S7, Supporting Information). Moreover, we also confirmed the design criteria of the NO release rate. The cGMP expression in platelets and SMCs resulting from Cu^II^-DA/HD coatings was proportional to the feeding concentration of CuCl_2_. When it increased to 12.5 *μ*g/mL, there was a significant inhibition of SMC attachment and proliferation. In contrast, the NO generation (with CuCl_2_ < 50 *μ*g/mL) stimulated the attachment, spreading, and proliferation of ECs. A favorable microenvironment should selectively promote EC growth advantage over SMCs. The importance of competitive capacity of ECs over SMCs instead of EC amount for pure endothelial layer development and better antirestenosis performance has been raised by Ji's lab [57]. This was achieved with NO release flux of 4.4 × 10^−10^ mol/cm^2^/min (CuCl_2_ feeding of 25 *μ*g/mL) and applied for all following animal tests. This finding was consistent with our previous studies, where NO generation coatings with NO fluxes over 5 × 10^−10^ mol/cm^2^/min exhibiting toxicity to ECs due to increased ROS production [36].

The influence of heparin on EC behaviors was reported to be dose-dependent. A density of heparin over 20 *μ*g/cm^2^ was not helpful in vascular cell proliferation; meanwhile, a lower density of 3.5 *μ*g/cm^2^ showed selective suppression on SMC proliferation but with promotion on endothelialization. Thus, the sensitivity to heparin density differs among vascular cells with higher density impairing all kinds of vascular cells, lower density selectively suppressing SMC hyperplasia but enhancing EPC and EC proliferation. Based on numerous heparinized surfaces, we conclude that materials modified with heparin, either covalently or noncovalently, result in different functions depending on the density of exposed heparin [35, 69]. These fundamental researches suggest potential of a multifunctional heparinized surface that could reduce restenosis and promote endothelialization, offering concept of more effective heparin application in surface modification. The QCM-D real-time monitoring data confirmed that the grafting amounts of heparin on Cu^II^-DA/HD coatings were proportional to the density of surface amine groups regulated by the initial CuCl_2_ concentration, which ranged from 1064 to 732 ng/cm^2^ (Figure S4, Supporting Information). The heparin conjugation density below 1 *μ*g/cm^2^ in this work enhanced the EC growth and reduced the SMC growth and thus provided a beneficial platform for the competitive growth of EC over SMC.

The present study established the combination of biologically derived coating chemistry by applying MCAN strategy for endothelium-mimicking engineering from surface heparin anchoring to interfacial NO catalytic release. The organization of Cu^2+^ ions in the MCAN imparts GPx-like activity of NO catalytic generation to the coating, while the primary amine groups on MCAN-based surface allow the covalent grafting of heparin. The anticoagulant performance of heparin depends on the combination with AT-III to inhibit coagulation factors, mainly thrombin and factor Xa. NO inhibits platelet aggregation by increasing the expression of cGMP, which makes NO a potent mediator in interrupting the clotting process seen by substantial inhibition of platelet activation *in vitro* and *in vivo* [36]. This coating exhibited significantly improved thromboresistivity compared with individual heparin immobilization or NO generation. The synergistic use of a multivalent strategy of EC-derived antiplatelet and anticoagulant agents therefore offered the best route to prevent thrombosis.

In summary, we developed here a metal-catechol-(amine) (MCA) strategy for fabricating multifunctional endothelium-mimicking coating on vascular stents. The multifunctional MCA coating with Cu^2+^ and abundant amine groups led to a combinatorial endothelium-like function with NO-generating catalytic activity and surface heparinization. As used for tailoring the surface of 316L SS cardiovascular stents, the coating exhibited a tunable NO generating rate and could last for 60 days at the physiological level of 2.2 × 10^−10^ mol/cm^2^/min. Together with the highly heparinized surface (1 *μ*g/cm^2^), the MCA coating could also exhibit enhanced antithrombogenicity, reduced inflammation, rapid endothelialization at the molecular level, and finally the prevention of in-stent restenosis *in vivo*. These results suggested that our MCA surface engineering strategy and the resultant endothelium-mimicking multifunctional coating could address critical clinical complications associated with cardiovascular devices and would also establish a versatile platform for bioengineering of metal implants.

## 4. Materials and Methods

### 4.1. Preparation of Cu^II^-DA/HD Coatings

The Cu^II^-DA/HD-coated 316L SS substrates and vascular stents were obtained by a one-step molecule/ion coassembled process. Dopamine hydrochloride (1 mg/mL) was mixed with HD (2.44 mg/mL) and CuCl_2_ with feeding concentration ranging from 0 to 50 *μ*g/mL in Tris buffer (1.2 mg/mL, pH 8.5) for 30 minutes for forming Cu^II^-DA/HD networks in solution. Afterward, the targeted objects were placed in the mixed solution for 48 h at 20°C, followed by thorough rinsing using distilled water to remove weakly bound polymers.

The coating thickness was measured through a spectroscopic ellipsometer (M-2000V, J.A. Woollam, USA). Cauchy's model was operated at a wavelength of 370-1000 nm with determined Δ and Ψ values. The density of the amine group on the Cu^II^-DA/HD films was detected by an AO II colorimetric method based on the standard curve of AO II [39]. The chemical composition was determined by XPS (XSAM800, Kratos Ltd., UK). Both wide and detailed XPS spectra of interested peaks (Cu2p3) were checked.

MALDI-TOF MS was recorded by a MALDI micro MX time-of-flight mass spectrometer (Waters, Milford, MA) using reflection mode. The detailed process has been reported elsewhere [39]. EPR measurement was performed to explore the role of the Cu^II^-DA/HD coordination in the coating formation. EPR spectra were obtained as reported before [28].

The morphology of Cu^II^-DA/HD-modified 316L SS stent (1.75 mm × 18 mm) was observed using field emission SEM (JSM-7001F, JEOL Ltd., Japan). The stent was expanded from 1.75 mm (diameter) to 3.0 mm under 8 atm with an angioplasty balloon.

### 4.2. Heparin Conjugation and Quantification

Heparin (1 mg/mL, Sigma-Aldrich) was preactivated through water-soluble carbodiimide (WSC, pH 5.5, consisting of 1 mg/mL *N*-(3-dimethylaminopropyl)-*N*′-ethylcarbodiimide (EDC, Sigma-Aldrich), 0.5 mg/mL *N*-hydroxysuccinimide (NHS, Sigma-Aldrich) and 10 mg/mL 2-(*N*-morpholino)ethanesulfonic acid hydrate (MES, Sigma-Aldrich)) solution for 15 min [39]. Afterward, the Cu^II^-DA/HD-modified substrates were placed in the activated heparin solution for 12 h, followed by rinsing with phosphate-buffered saline (PBS) and distilled water. The chemical composition of the heparin-conjugated surfaces was analyzed by XPS as described.

The amount of conjugated heparin was real-time monitored by a QCM-D (Q-sense AB, Sweden). Cu^II^-DA/HD was formed on the AT-cut 5 MHz Au with quartz crystal coating (diameter of 10 mm). The information of resonance frequencies (Δ*F*) and relaxation (Δ*D*) of the vibration was recorded. In brief, the Cu^II^-DA/HD-modified quartz crystal was placed in the QCM chamber with MES buffer (pH 5.4) continuously flowing in at 50 *μ*L/min until the QCM traces reached balance. Heparin solution (1 mg/mL, pH 5.4) was then flowing through the measurement chamber until the curve reached equilibrium. Afterward, the PBS solution was applied to move free heparin. The final adsorbed heparin mass (Δ*m*) was calculated based on Sauerbrey equation [70]:

(1) $$\begin{array}{c} \Delta m=-\frac{C\cdot \Delta f}{n}, \end{array}$$

where *C* stands for a constant indicating the sensitivity to mass changes and *n* stands for the overtone number.

Heparin bioactivity was detected through anti-FXa test that reflects the binding efficiency of ATIII to Fxa [71]. In the presence of heparin, the formed heparin-ATIII complexes bind with FXa, decreasing the residual FXa to react with the chromogenic substrate and ultimately leading to a decrease in absorbance. The samples were firstly incubated with 50 *μ*L platelet-poor plasma (PPP) (in PBS as a volume ratio of 1 : 4) at 37°C for 30 min. Afterward, the PPP was removed and 50 *μ*L S2732 (preheated at 37°C) with 50 *μ*L Factor Xa was added. After incubation for 2 min, 100 *μ*L of supernatant was taken to a new 96-well plate from each sample. Acetic acid was introduced to stop the reaction, and measurement at 405 nm (absorbance) was performed using a microplate reader.

### 4.3. Nitric Oxide Catalytic Release

The NO generation from Cu^II^-DA/HD and Hep@NO samples was tested using an NO analyzer (NOA, Seivers 280i, Boulder, CO). Samples were immersed in the test solution consisting of 10 *μ*M GSH and 10 *μ*M GSNO. The generated NO gas was diffused in the test solution and delivered to an NO analyzer through N_2_(g) stream. The amount of generated NO was calculated depending on the standard curves reported elsewhere [25].

### 4.4. Stability Test

To test the longevity of NO generation and heparin bioactivity, the Hep@NO-coated samples were continuously exposed to the PBS solution containing 10 *μ*M GSh and 10 *μ*M GSNO for various periods (medium changed every 12 h). After 2, 7, 15, 30, and 60 days, the NO catalytic experiments, XPS, and anti-FXa assay were performed accordingly.

### 4.5. In Vitro Hemocompatibility Evaluation

Fresh human blood was acquired from the Blood Centre of Chengdu, China, according to ethics rules. The experiments in this study were conducted within 12 h after donation. PPP was obtained through blood centrifugation with a speed of 3000 rpm for 15 min, while PRP was obtained with a speed of 1500 rpm for 15 min.

The Fg adsorption test was performed by adding PPP (50 *μ*L) onto samples at 37°C. After 2 hours, sample was washed with PBS; then 20 *μ*L of HRP-labeled sheep PAb anti-human fibrinogen (Sigma-Aldrich) was introduced and incubated for 60 minutes. Then, chromogenic substrate 3,3′,5,5′-tetramethylbenzidine solution (TMB, 100 *μ*L) was added. After 10 minutes, the color reaction was terminated by introducing acid solution, while measurement at 405 nm (absorbance) was performed. In terms of Fg activation, the samples were firstly incubated with PPP for 120 min, then washed using PBS and incubated with mouse anti-human *γ*-fibrinogen monoclonal antibody (20 *μ*L) at 37°C. After one hour, the sample was washed and reacted with HRP-labeled sheep anti-mouse polyclonal antibody (20 *μ*L). Afterward, TMB solution (100 *μ*L) was introduced and incubated for 10 min. Measurement at 405 nm (absorbance) was performed following acid solution supplement [72].

Platelet adhesion assay was conducted by incubating PRP (50 *μ*L) on samples. After 2 h, samples were fully rinsed in PBS and fixed by 2.5% glutaraldehyde solution for overnight, followed by gradient dehydrated and dealcoholized. The morphology of samples was determined by SEM. The expression of P-selectin (platelet activation marker) was detected as reported elsewhere [72]. cGMP expressions were determined using human cGMP ELISA kit. Samples were immersed in PRP (1 mL, with 10 *μ*M GSNO and 10 *μ*M GSH) for 30 min. Triton-X was then introduced (10%, 100 *μ*L) with sonication following. The mixture was collected and centrifuged at 2500 rpm, and the supernatant was collected and analyzed following the instruction of the cGMP ELISA kit.

### 4.6. Smooth Muscle Cell Culture and Evaluation

HUASMCs were obtained as reported before [72]. Cells used in this study were between the 2^nd^ and 5^th^ passages with a density of 5 × 10^4^ cells/cm^2^. After incubation for 2 h, 1 day, and 3 days, samples were harvested and fixed in 4% paraformaldehyde (PFA) for 30 min. Cell staining was performed following the instruction as reported before [72]. The RSNO donor solution was added every 4 hours. Cell proliferation was tested using CCK-8 kit after 1 and 3 days, respectively. Briefly, 350 *μ*L fresh culture medium with 10% CCK-8 reagent was replaced. After 3 hours, measurement at 450 nm (absorbance) was performed. The RSNO donor solution was added every 4 hours. cGMP was analyzed after 2 h incubation following human cGMP ELISA kit instruction.

### 4.7. Endothelium Cell Culture and Evaluation

HUVECs were harvested from human umbilical veins as reported before [72]. Cells used in this study were in the 1^st^ passage with a density of 5 × 10^4^ cells/cm^2^. After incubation for 2 h, 1 day, and 3 days, samples were washed and fixed with 4% PFA for 30 min. For phalloidin staining, samples were firstly treated with bovine serum albumin (1%, Sigma-Aldrich) for 1 h, then incubated in FITC labeled phalloidin (1 : 50 in PBS, Sigma-Aldrich) for another hour. 4′,6-Diamidino-2-phenylindole (DAPI) was applied as a counterstain. Laser scanning confocal microscopy was used for cell morphology observation. Cell proliferation was evaluated using CCK-8 kit with the time points of 1 and 3 days, respectively. RSNO donor solution was added every 4 hours.

Total ribonucleic acid (RNA) was collected by a TRIzol reagent (Life Technologies) for Real-Time polymerase chain reaction (RT-qPCR). In brief, complementary DNA (cDNA) was synthesized using 1,000 ng of harvested RNA following the instruction of SensiFAST™ cDNA Synthesis Kit (Bioline, Australia). The mRNA expressions of platelet/endothelial cell adhesion molecule 1 (PECAM, also known as CD31), vWF, eNOS, and FGF were tested using QuantStudio™ RT-qPCR equipment (Applied Biosystems) through SYBR Green qPCR Master Mix (Life Technologies). Data were calculated following comparative CT (*ΔΔ*CT) mode and listed as relative RNA levels after normalization to glyceraldehyde 3-phosphate dehydrogenase (GAPDH). The detailed list of primer sequences can be found in Table S3.

### 4.8. Coculture of ECs and SMCs

HUVECs (green chloromethyl fluorescein diacetate (CMFDA)) and HUASMCs (orange chloromethyl trimethyl rhodamine (CMTMR)) were prelabelled in this assay [39]. Cell suspensions were mixed with a volume ratio of 1 : 1 (5 × 10^4^ cells/cm^2^), respectively. The coculture cell adhesion and proliferation were inspected after 2 and 24 hours using a fluorescence microscope. The quantities of grown cells were calculated with no less than 12 images of each group.

### 4.9. Macrophage Response to the Engineered Surface

Macrophage RAW 264.7 cells were seeded on samples with a density of 5 × 10^4^ cells/cm^2^. After incubation for 1 day, all samples were washed with PBS and fixed in glutaraldehyde solution (2.5 wt%). Rhodamine123 fluorescent staining was followed, and cellular morphology was inspected using a fluorescence microscope. RSNO donor supplement was added every 4 hours. The expression levels of the target mRNA (inflammatory cytokines: TNF-*α*, IL-6, IL-18, and IL-1*β*; macrophage-phenotype markers: CD86, iNOS, CD206, CD163, and IL-10) resulting from treated M*Φ* were tested by RT-qPCR as described. The detailed list of primer sequences can be found in Table S3.

### 4.10. Ex Vivo Circulation Thrombogenicity Test

ECC was conducted by connecting the left carotid artery of New Zealand white rabbits (6, 2.5~3.5 kg) with the right jugular vein in accordance with guidelines as reported before [51]. Samples prepared on 316L SS foils (0.8 × 1 mm^2^) were rolled and installed in the tube. After 2 h, the blood circuit was terminated and cross-sessional photographs of tube installed with samples were taken for tube occlusion calculation. The blood flow rate was monitored during the whole process. Afterward, samples were carefully removed from ECC. The attached thrombi were collected, inspected, and weighted. Samples were then fully rinsed with PBS and fixed by glutaraldehyde solution (2.5%) for overnight. After gradient dehydration and drying, samples were inspected by SEM.

### 4.11. In Vivo Stent Implantation

28 adult New Zealand White Rabbits (2.5-3.5 kg) and 56 stents were applied in stent implantation. There were two group of stents involved, control 316L SS stent and Hep@NO modified ones. One stent from each group was bilaterally placed in the left and right iliac arteries of rabbits, respectively [10]. To evaluate the antithrombogenic properties of stents, the vessels with the implanted stent were harvested and cut open lengthwise, followed by fixation for observation by SEM after 2 h of implantation.

To assess the endothelialization levels of different stents, immunofluorescence images were taken after the implantation of one week. In brief, the vessels with the implanted stent were harvested and cut open lengthwise, followed by fixation in 4% PFA for 30 min. After Triton X-100 (0.5%, PBS, 30 min) treatment and 1% BSA blocking overnight, the segments reacted with mouse anti-rabbit CD31 antibody (1 : 300 in PBS, Novus Biologicals) and Alexa Fluor® 488-conjugated goat anti-mouse IgG secondary antibody (1 : 800 in PBS, Absin Bioscience), each for 6 hours. Afterward, DAPI and FITC-labeled phalloidin (1 : 50 in PBS, Sigma-Aldrich) were used for nuclear counterstaining and F-actin staining, respectively. Laser scanning confocal microscopy (green: CD31, red: F-actin, blue: cell nucleus) was then used for cell morphology observation, and fluorescence intensities along the stent strut were detected.

In terms of 1- and 3-month groups, half of the collected stenting arteries were fixed for SEM observation. Another half were fixed for tissue histological staining. Resin-embedded tissue cross-sections were then stained for Van Gieson. Images were captured using a histological microscope. The mean neointimal area as well as percent neointimal stenosis analysis were performed based on the histomorphometric images. The stented arteries were also collected for target gene expression after one month. RNA was isolated from the artery followed by grinding using a homogenizer and extraction by a TRIzol reagent (Life Technologies). The expression levels of the target mRNA (CD31, vWF, eNOS, *α*-SMA, CRBP-1, PCNA, IL-6, TNF-*α*, and IL-10) were determined by RT-qPCR as described. The detailed list of primer sequences can be found in Table S3.

### 4.12. Statistical Analysis

All data in this study are exhibited as the mean ± standard deviation. Statistical analysis was conducted by applying SPSS software, employing a one-way ANOVA as detailed in the figure captions. Tests that have an alpha level for significance set at *p* < 0.05 were considered significant difference. All of the tests were performed at least thrice with no less than four parallel samples.

## Data Availability

The data that support the findings of this study are available from the corresponding author on request.

## Supplementary Materials

Supplementary Materials Figure S1: mechanical properties of Cu^II^-DA/HD coating. Scanning electron microscope (SEM) graphs of the 316L SS stent before (A) and after dilatation (B). (C) Cu^II^-DA/HD-coated SS stent was mounted onto an angioplasty balloon and dilated. (D) SEM graphs of the 316L SS stent coated with Cu^II^-DA/HD after dilatation. DA: dopamine, HD: hexamethylenediamine. Figure S2: X-ray photoelectron spectroscopy XPS spectra of Cu^II^-DA/HD coated 316L SS substrates (for [DA] and [HD] constant at 1 mg/mL and 2.44 mg/mL, respectively, Cu^II^-DA/HD coatings were obtained over a [CuCl_2_] range from 0 to 50 *μ*g/mL). The content of copper was a function of the amount of CuCl_2_ that was added in the reaction solution used for deposition of the Cu^II^-DA/HD coatings (see Table S1). Figure S3: electron paramagnetic resonance (EPR) and matrix-assisted laser desorption ionization mass spectrometry (MALDI-MS) analysis of Cu^II^-DA/HD coating, suggested reaction mechanism, molecule/ion coassembly, and copper-catechol-(amine) network formation mechanisms. EPR (A) and MALDI MS (B) analysis of Cu^II^-DA/HD coating prepared in Tris buffer (pH 8.5). (C) Suggested molecule/ion coassembly mechanism of Cu^2+^, DA, and HD based on EPR and MALDI MS analysis. Figure S4: amount of covalently immobilized heparin on Cu^II^-DA/HD coatings ([DA] and [HD] constant at 1 mg/mL and 2.44 mg/mL, respectively, Cu^II^-DA/HD coatings were obtained over a [CuCl_2_] range from 0 to 50 *μ*g/mL). Real-time monitoring by QCM-D, mean ± SD (*n* = 4). Figure S5: XPS spectra of Cu^II^-DA/HD coatings ([DA] and [HD] constant at 1 mg/mL and 2.44 mg/mL, respectively, Cu^II^-DA/HD coatings were obtained over a [CuCl_2_] range from 0 to 50 *μ*g/mL) after grafting heparin. (A) XPS wide scans and (B) evolution of the S content determined from the XPS spectra of the Cu^II^-DA/HD coatings grafted with heparin. Figure S6: anti-FXa activity of the heparin on the Cu^II^-DA/HD coatings ([DA] and [HD] constant at 1 mg/mL and 2.44 mg/mL, respectively, Cu^II^-DA/HD coatings were obtained over a [CuCl_2_] range from 0 to 50 *μ*g/mL). Figure S7: evolution of the catalytic release of nitric oxide (NO) from the Cu^II^-DA/HD coatings without (A) and with (B) grafted heparin. Data presented as the mean ± SD (*n* = 4). Figure S8: proliferation of HUASMCs. The NO donor solution (10 *μ*M GSNO, 10 *μ*M GSH) was added to the cell culture medium every 4 hours for mimicking the in vivo blood environment. Figure S9: the proliferation of HUVECs. The NO donor solution (10 *μ*M GSNO, 10 *μ*M GSH) was added to the cell culture medium every 4 hours for mimicking the in vivo blood environment. Figure S10: the fluorescence staining images of HUVECs on bare 316L SS, Hep, NO, and Hep@NO coatings after 3 days of culture (green: actin, blue: cell nuclei). Figure S11: (A) images of cocultured ECs/SMCs (HUVECs (green) and HUASMCs (red)) on samples after 2 hours. Figure S12: adhesion and cGMP synthesis of platelets. The NO donor solution (10 *μ*M GSNO, 10 *μ*M GSH) was added to platelet-rich plasma for better mimicking the in vivo blood environment. Figure S13: the fluorescence intensities of different components along the line segments in the images of stented-vessel shown in Figure 8(a) (blue: cell nucleus, red: F-actin, green: CD31). Figure S14: stent implantation *in vivo*. (A) Reendothelialization of Hep@NO-coated and bare stents after 1 and 3 months of implantation evaluated by SEM. Table S1: chemical compositions of the Cu^II^-DA/HD coatings fabricated using CuCl_2_ with feed concentrations ranging from 0 to 50 *μ*g/mL. Table S2: chemical compositions of the Cu^II^-DA/HD coatings after grafting of heparin. Table S3: RNA primers applied in this study.

## References

[1] Benjamin E. J., Virani S. S., Callaway C. W. (2018). Heart disease and stroke statistics&-2018 update: a report from the American Heart Association. *Circulation*.

[2] Rauch U., Osende J. I., Fuster V., Badimon J. J., Fayad Z., Chesebro J. H. (2001). Thrombus formation on atherosclerotic plaques: pathogenesis and clinical consequences. *Annals of Internal Medicine*.

[3] McCormick C., Wall J. G., Podbielska H., Wawrzyńska M. (2018). 1 - Overview of cardiovascular stent designs. *Functionalised Cardiovascular Stents*.

[4] Douglas G., van Kampen E., Hale A. B. (2013). Endothelial cell repopulation after stenting determines in-stent neointima formation: effects of bare-metal vs. drug-eluting stents and genetic endothelial cell modification. *European Heart Journal*.

[5] McHugh P., Barakat A., McGinty S. (2016). Medical stents: state of the art and future directions. *Annals of Biomedical Engineering*.

[6] Lansky A., Wijns W., Xu B. (2018). Targeted therapy with a localised abluminal groove, low-dose sirolimus- eluting, biodegradable polymer coronary stent (TARGET All Comers): a multicentre, open-label, randomised non-inferiority trial. *The Lancet*.

[7] Saito S., Krucoff M. W., Nakamura S. (2018). Japan-United States of America Harmonized Assessment by Randomized Multicentre Study of OrbusNEich’s Combo StEnt (Japan-USA HARMONEE) study: primary results of the pivotal registration study of combined endothelial progenitor cell capture and drug-eluting stent in patients with ischaemic coronary disease and non-ST-elevation acute coronary syndrome. *European Heart Journal*.

[8] Ylä-Herttuala S., Martin J. F. (2000). Cardiovascular gene therapy. *The Lancet*.

[9] Kojima Y., Volkmer J. P., McKenna K. (2016). CD47-blocking antibodies restore phagocytosis and prevent atherosclerosis. *Nature*.

[10] Yang Z., Yang Y., Zhang L. (2018). Mussel-inspired catalytic selenocystamine-dopamine coatings for long-term generation of therapeutic gas on cardiovascular stents. *Biomaterials*.

[11] Hauser S., Jung F., Pietzsch J. (2017). Human endothelial cell models in biomaterial research. *Trends in Biotechnology*.

[12] O’Connor I. B., Wall J. G., Wall J. G., Podbielska H., Wawrzyńska M. (2018). 16 - Immobilization of antibodies on cardiovascular stents. *Functionalised Cardiovascular Stents*.

[13] Jansen F., Li Q., Pfeifer A., Werner N. (2017). Endothelial- and immune cell-derived extracellular vesicles in the regulation of cardiovascular health and disease. *JACC: Basic to Translational Science*.

[14] Ganz P., Hsue P. Y. (2013). Endothelial dysfunction in coronary heart disease is more than a systemic process. *European Heart Journal*.

[15] Jourde-Chiche N., Fakhouri F., Dou L. (2019). Endothelium structure and function in kidney health and disease. *Nature Reviews Nephrology*.

[16] Gresele P., Momi S., Guglielmini G. (2019). Nitric oxide-enhancing or -releasing agents as antithrombotic drugs. *Biochemical Pharmacology*.

[17] Pober J. S., Sessa W. C. (2007). Evolving functions of endothelial cells in inflammation. *Nature Reviews Immunology*.

[18] Park K.-H., Park W. J. (2015). Endothelial dysfunction: clinical implications in cardiovascular disease and therapeutic approaches. *Journal of Korean Medical Science*.

[19] Zhang F., Zhang Q., Li X., Huang N., Zhao X., Yang Z. (2019). Mussel-inspired dopamine-Cu<sup>II</sup> coatings for sustained in situ generation of nitric oxide for prevention of stent thrombosis and restenosis. *Biomaterials*.

[20] Tu Q., Shen X., Liu Y. (2019). A facile metal–phenolic–amine strategy for dual-functionalization of blood-contacting devices with antibacterial and anticoagulant properties. *Materials Chemistry Frontiers*.

[21] Qiu H., Qi P., Liu J. (2019). Biomimetic engineering endothelium-like coating on cardiovascular stent through heparin and nitric oxide-generating compound synergistic modification strategy. *Biomaterials*.

[22] Yang Y., Qi P. K., Yang Z. L., Huang N. (2015). Nitric oxide based strategies for applications of biomedical devices. *Biosurface and Biotribology*.

[23] Kouretas P. C., Hannan R. L., Kapur N. K. (1998). Non-anticoagulant heparin increases endothelial nitric oxide synthase activity: role of inhibitory guanine nucleotide proteins. *Journal of Molecular and Cellular Cardiology*.

[24] Dandona P., Qutob T., Hamouda W., Bakri F., Aljada A., Kumbkarni Y. (1999). Heparin inhibits reactive oxygen species generation by polymorphonuclear and mononuclear leucocytes. *Thrombosis Research*.

[25] Cha W., Meyerhoff M. E. (2007). Catalytic generation of nitric oxide from S-nitrosothiols using immobilized organoselenium species. *Biomaterials*.

[26] Carpenter A. W., Schoenfisch M. H. (2012). Nitric oxide release: part II. Therapeutic applications. *Chemical Society Reviews*.

[27] Ren X., Feng Y., Guo J. (2015). Surface modification and endothelialization of biomaterials as potential scaffolds for vascular tissue engineering applications. *Chemical Society Reviews*.

[28] Li X., Qiu H., Gao P., Yang Y., Yang Z., Huang N. (2018). Synergetic coordination and catecholamine chemistry for catalytic generation of nitric oxide on vascular stents. *NPG Asia Materials*.

[29] Luo R., Zhang J., Zhuang W. (2018). Multifunctional coatings that mimic the endothelium: surface bound active heparin nanoparticles within situgeneration of nitric oxide from nitrosothiols. *Journal of Materials Chemistry B*.

[30] Simon-Walker R., Romero R., Staver J. M. (2017). Glycocalyx-inspired nitric oxide-releasing surfaces reduce platelet adhesion and activation on titanium. *ACS Biomaterials Science & Engineering*.

[31] Naghavi N., de Mel A., Alavijeh O. S., Cousins B. G., Seifalian A. M. (2013). Nitric oxide donors for cardiovascular implant applications. *Small*.

[32] Wu B., Gerlitz B., Grinnell B. W., Meyerhoff M. E. (2007). Polymeric coatings that mimic the endothelium: combining nitric oxide release with surface-bound active thrombomodulin and heparin. *Biomaterials*.

[33] Zhou Z., Meyerhoff M. E. (2005). Preparation and characterization of polymeric coatings with combined nitric oxide release and immobilized active heparin. *Biomaterials*.

[34] Helft G. (2016). Dual antiplatelet therapy duration after drug-eluting stents: how long?. *Journal of Thoracic Disease*.

[35] Gong F., Cheng X., Wang S., Zhao Y., Gao Y., Cai H. (2010). Heparin-immobilized polymers as non-inflammatory and non-thrombogenic coating materials for arsenic trioxide eluting stents. *Acta Biomaterialia*.

[36] Yang Z., Yang Y., Xiong K., Wang J., Lee H., Huang N. (2018). Metal-phenolic surfaces for generating therapeutic nitric oxide gas. *Chemistry of Materials*.

[37] Yang Z., Tu Q., Wang J., Huang N. (2012). The role of heparin binding surfaces in the direction of endothelial and smooth muscle cell fate and re-endothelialization. *Biomaterials*.

[38] Stewart E. M., Liu X., Clark G. M., Kapsa R. M. I., Wallace G. G. (2012). Inhibition of smooth muscle cell adhesion and proliferation on heparin-doped polypyrrole. *Acta Biomaterialia*.

[39] Yang Y., Qi P., Wen F. (2014). Mussel-inspired one-step adherent coating rich in amine groups for covalent immobilization of heparin: hemocompatibility, growth behaviors of vascular cells, and tissue response. *ACS Applied Materials & Interfaces*.

[40] Park J., Pramanick S., Park D. (2017). Therapeutic-gas-responsive hydrogel. *Advanced Materials*.

[41] Lertkiatmongkol P., Liao D., Mei H., Hu Y., Newman P. J. (2016). Endothelial functions of platelet/endothelial cell adhesion molecule-1 (CD31). *Current Opinion in Hematology*.

[42] Yang X., Liaw L., Prudovsky I. (2015). Fibroblast growth factor signaling in the vasculature. *Current Atherosclerosis Reports*.

[43] Lavender M. D., Pang Z., Wallace C. S., Niklason L. E., Truskey G. A. (2005). A system for the direct co-culture of endothelium on smooth muscle cells. *Biomaterials*.

[44] Lusis A. J. (2000). Atherosclerosis. *Nature*.

[45] Davis C., Fischer J., Ley K., Sarembock I. J. (2003). The role of inflammation in vascular injury and repair. *Journal of Thrombosis and Haemostasis*.

[46] Mosser D. M., Edwards J. P. (2008). Exploring the full spectrum of macrophage activation. *Nature Reviews Immunology*.

[47] Li Y., Xiao Y., Liu C. (2017). The horizon of materiobiology: a perspective on material-guided cell behaviors and tissue engineering. *Chemical Reviews*.

[48] Lee W. J., Tateya S., Cheng A. M. (2015). M2 macrophage polarization mediates anti-inflammatory effects of endothelial nitric oxide signaling. *Diabetes*.

[49] Wang Z., Cui Y., Wang J. (2014). The effect of thick fibers and large pores of electrospun poly(*ε*-caprolactone) vascular grafts on macrophage polarization and arterial regeneration. *Biomaterials*.

[50] Davie E. W., Fujikawa K. (1975). Basic mechanisms in blood coagulation. *Annual Review of Biochemistry*.

[51] Yang Y., Li X., Qiu H. (2018). Polydopamine modified TiO2Nanotube arrays for long-term controlled elution of bivalirudin and improved hemocompatibility. *ACS Applied Materials & Interfaces*.

[52] Farah C., Michel L. Y. M., Balligand J. L. (2018). Nitric oxide signalling in cardiovascular health and disease. *Nature Reviews Cardiology*.

[53] Liu K., Jiang L. (2011). Bio-inspired design of multiscale structures for function integration. *Nano Today*.

[54] Sun T., Qing G., Su B., Jiang L. (2011). Functional biointerface materials inspired from nature. *Chemical Society Reviews*.

[55] Castro-Ferreira R., Cardoso R., Leite-Moreira A., Mansilha A. (2018). The role of endothelial dysfunction and inflammation in chronic venous disease. *Annals of Vascular Surgery*.

[56] Danese S., Dejana E., Fiocchi C. (2007). Immune regulation by microvascular endothelial cells: directing innate and adaptive immunity, coagulation, and inflammation. *The Journal of Immunology*.

[57] Wei Y., Ji Y., Xiao L.-L. (2013). Surface engineering of cardiovascular stent with endothelial cell selectivity for in vivo re-endothelialisation. *Biomaterials*.

[58] Chang H., Hu M., Zhang H. (2016). Improved endothelial function of endothelial cell monolayer on the soft polyelectrolyte multilayer film with matrix-bound vascular endothelial growth factor. *ACS Applied Materials & Interfaces*.

[59] Lin Q., Ding X., Qiu F., Song X., Fu G., Ji J. (2010). In situ endothelialization of intravascular stents coated with an anti-CD34 antibody functionalized heparin–collagen multilayer. *Biomaterials*.

[60] Dane M. J., van den Berg B. M., Lee D. H. (2015). A microscopic view on the renal endothelial glycocalyx. *American Journal of Physiology-Renal Physiology*.

[61] Reitsma S., Slaaf D. W., Vink H., van Zandvoort M. A. M. J., oude Egbrink M. G. A. (2007). The endothelial glycocalyx: composition, functions, and visualization. *Pflügers Archiv - European Journal of Physiology*.

[62] Weinbaum S., Tarbell J. M., Damiano E. R. (2007). The structure and function of the endothelial glycocalyx layer. *Annual Review of Biomedical Engineering*.

[63] Hunt A. P., Batka A. E., Hosseinzadeh M. (2019). Nitric oxide generation on demand for biomedical applications via electrocatalytic nitrite reduction by copper BMPA- and BEPA-carboxylate complexes. *ACS Catalysis*.

[64] Konopińska K. K., Schmidt N. J., Hunt A. P. (2018). Comparison of copper(II)–ligand complexes as mediators for preparing electrochemically modulated nitric oxide-releasing catheters. *ACS Applied Materials & Interfaces*.

[65] Lutzke A., Pegalajar-Jurado A., Neufeld B. H., Reynolds M. M. (2014). Nitric oxide-releasing S-nitrosated derivatives of chitin and chitosan for biomedical applications. *Journal of Materials Chemistry B*.

[66] Chen Z., Klein T., Murray R. Z. (2016). Osteoimmunomodulation for the development of advanced bone biomaterials. *Materials Today*.

[67] Gao J., Jiang L., Liang Q. (2018). The grafts modified by heparinization and catalytic nitric oxide generation used for vascular implantation in rats. *Regenerative Biomaterials*.

[68] Alayash A. I. (2004). Oxygen therapeutics: can we tame haemoglobin?. *Nature Reviews Drug Discovery*.

[69] Ding Y., Yang Z., Bi C. W. C. (2014). Directing vascular cell selectivity and hemocompatibility on patterned platforms featuring variable topographic geometry and size. *ACS Applied Materials & Interfaces*.

[70] Marx K. A. (2003). Quartz crystal microbalance: a useful tool for studying thin polymer films and complex biomolecular systems at the solution−surface interface. *Biomacromolecules*.

[71] Maitz M. F., Freudenberg U., Tsurkan M. V., Fischer M., Beyrich T., Werner C. (2013). Bio-responsive polymer hydrogels homeostatically regulate blood coagulation. *Nature Communications*.

[72] Yang Z., Tu Q., Maitz M. F., Zhou S., Wang J., Huang N. (2012). Direct thrombin inhibitor-bivalirudin functionalized plasma polymerized allylamine coating for improved biocompatibility of vascular devices. *Biomaterials*.

