# Supplementary material for: Endothelium-Mimicking Multifunctional Coating Modified Cardiovascular Stents via a Stepwise Metal-Catechol-(Amine) Surface Engineering Strategy
Source: Research (Wash D C). 2020 Apr 24;2020:9203906. doi: 10.34133/2020/9203906 (PMC7196174; doi:10.34133/2020/9203906)
Supplement: Supplementary Materials — Figure S1: mechanical properties of CuII-DA/HD coating. Scanning electron microscope (SEM) graphs of the 316L SS stent before (A) and after dilatation (B). (C) CuII-DA/HD-coated SS stent was mounted onto an angioplasty balloon and dilated. (D) SEM graphs of the 316L SS stent coated with CuII-DA/HD after dilatation. DA: dopamine, HD: hexamethylenediamine. Figure S2: X-ray photoelectron spectroscopy XPS spectra of CuII-DA/HD coated 316L SS substrates (for [DA] and [HD] constant at 1 mg/mL and 2.44 mg/mL, respectively, CuII-DA/HD coatings were obtained over a [CuCl2] range from 0 to 50 μg/mL). The content of copper was a function of the amount of CuCl2 that was added in the reaction solution used for deposition of the CuII-DA/HD coatings (see Table S1). Figure S3: electron paramagnetic resonance (EPR) and matrix-assisted laser desorption ionization mass spectrometry (MALDI-MS) analysis of CuII-DA/HD coating, suggested reaction mechanism, molecule/ion coassembly, and copper-catechol-(amine) network formation mechanisms. EPR (A) and MALDI MS (B) analysis of CuII-DA/HD coating prepared in Tris buffer (pH 8.5). (C) Suggested molecule/ion coassembly mechanism of Cu2+, DA, and HD based on EPR and MALDI MS analysis. Figure S4: amount of covalently immobilized heparin on CuII-DA/HD coatings ([DA] and [HD] constant at 1 mg/mL and 2.44 mg/mL, respectively, CuII-DA/HD coatings were obtained over a [CuCl2] range from 0 to 50 μg/mL). Real-time monitoring by QCM-D, mean ± SD (n = 4). Figure S5: XPS spectra of CuII-DA/HD coatings ([DA] and [HD] constant at 1 mg/mL and 2.44 mg/mL, respectively, CuII-DA/HD coatings were obtained over a [CuCl2] range from 0 to 50 μg/mL) after grafting heparin. (A) XPS wide scans and (B) evolution of the S content determined from the XPS spectra of the CuII-DA/HD coatings grafted with heparin. Figure S6: anti-FXa activity of the heparin on the CuII-DA/HD coatings ([DA] and [HD] constant at 1 mg/mL and 2.44 mg/mL, respectively, CuII-DA/HD coatings were ob [file 9203906.f1.docx]

Supplementary Materials

Endothelium-Mimicking Multifunctional Coating Modified Cardiovascular Stents via a Stepwise Metal-Catechol-(Amine) Surface Engineering Strategy

Ying Yang^1,2,3^, Peng Gao^1^, Juan Wang^1^, Qiufen Tu^1^, Long Bai^2,3^, Kaiqin Xiong^1^, Hua Qiu^1^, Xin Zhao^4^, Manfred F. Maitz^1,5^, Huaiyu Wang^6^, Xiangyang Li^1^, Qiang Zhao^7^, Yin Xiao^2,3^, Nan Huang^1,^* and Zhilu Yang^1,^*

^1^Key Laboratory of Advanced Technologies of Materials, Ministry of Education, School of Materials Science and Engineering, Southwest Jiaotong University, Chengdu, 610031, P. R. China.

^2^Institute of Health and Biomedical Innovation, Queensland University of Technology, Brisbane, 4059, Australia.

^3^Australia-China Centre for Tissue Engineering and Regenerative Medicine, Queensland University of Technology, Brisbane, 4059, Australia.

^4^Department of Biomedical Engineering, The Hong Kong Polytechnic University, Hung Hom, Kowloon, Hong Kong, P. R. China.

^5^Max Bergmann Center of Biomaterials, Leibniz Institute of Polymer Research Dresden, Hohe Strasse 6, 01069 Dresden, Germany.

^6^Institute of Biomedicine and Biotechnology, Shenzhen Institutes of Advanced Technology, Chinese Academy of Sciences, Shenzhen, 518055, P. R. China

^7^State Key Laboratory of Medicinal Chemical Biology, Key Laboratory of Bioactive Materials, Ministry of Education, College of Life Sciences, Nankai University, Tianjin, P. R. China

Correspondence should be addressed to Nan Huang; huangnan1956@163.com and Zhilu Yang; zhiluyang1029@swjtu.edu.cn

**
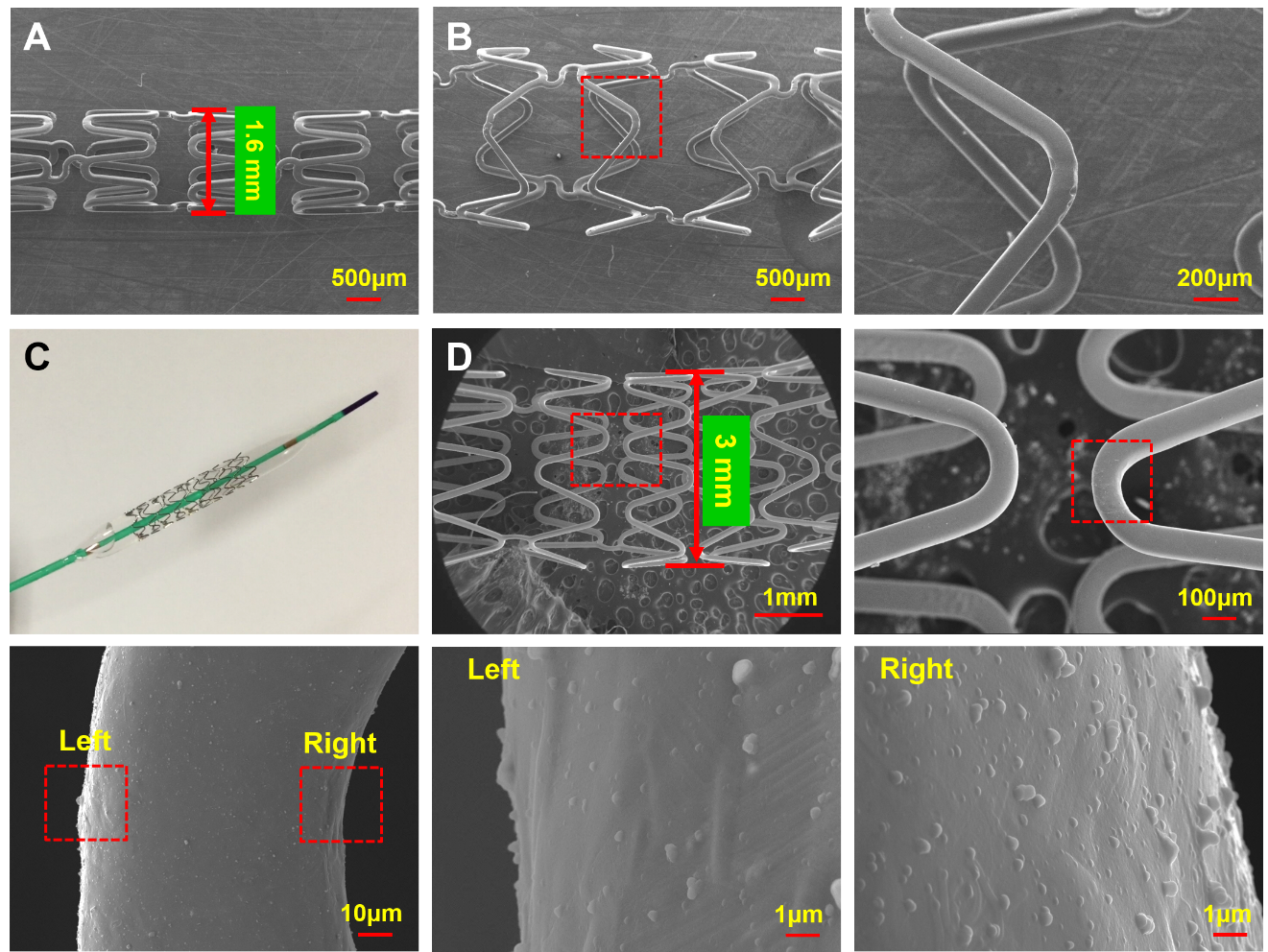
**

**Figure S1.** Mechanical properties of Cu^II^-DA/HD coating. [Scanning](file:///C:\Users\Yangy20\AppData\Local\youdao\dict\Application\7.5.0.0\resultui\dict\?keyword=scanning)[electron](file:///C:\Users\Yangy20\AppData\Local\youdao\dict\Application\7.5.0.0\resultui\dict\?keyword=electron)[microscope](file:///C:\Users\Yangy20\AppData\Local\youdao\dict\Application\7.5.0.0\resultui\dict\?keyword=microscope) (SEM) graphs of the 316L SS stent before (A) and after dilatation (B). (C) Cu^II^-DA/HD coated SS stent was mounted onto an angioplasty balloon and dilated. (D) SEM graphs of the 316L SS stent coated with Cu^II^-DA/HD after dilatation. DA: dopamine, HD: hexamethylenediamine.

**Figure S2.** X-ray photoelectron spectroscopy XPS spectra of Cu^II^–DA/HD coated 316L SS substrates (for [DA] and [HD] constant at 1 mg/mL and 2.44 mg/mL, respectively, Cu^II^–DA/HD coatings were obtained over a [CuCl_2_] range from 0 to 50 µg/mL). The content of copper was a function of the amount of CuCl_2_ that was added in the reaction solution used for deposition of the Cu^II^–DA/HD coatings (see **Table S1**).

**Figure S3.** Electron paramagnetic resonance (EPR) and matrix-assisted laser desorption ionization mass spectrometry (MALDI-MS) analysis of Cu^II^–DA/HD coating, suggested reaction mechanism, molecule/ion co-assembly, and copper-catechol-(amine) network formation mechanisms. EPR (A) and MALDI MS (B) analysis of Cu^II^–DA/HD coating prepared in Tris buffer (pH 8.5). (C) Suggested molecule/ion co-assembly mechanism of Cu^2+^, DA and HD based on EPR and MALDI MS analysis.

**Figure S4.** Amount of covalently immobilized heparin on Cu^II^–DA/HD coatings ([DA] and [HD] constant at 1 mg/mL and 2.44 mg/mL respectively, Cu^II^–DA/HD coatings were obtained over a [CuCl_2_] range from 0 to 50 µg/mL). Real-time monitoring by QCM-D, mean ± SD (n=4).


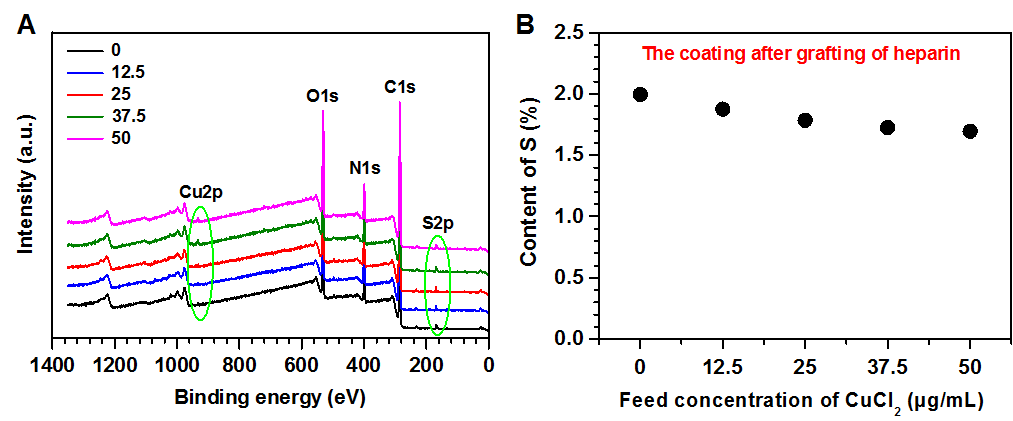


**Figure S5.** XPS spectra of Cu^II^–DA/HD coatings ([DA] and [HD] constant at 1 mg/mL and 2.44 mg/mL respectively, Cu^II^–DA/HD coatings were obtained over a [CuCl_2_] range from 0 to 50 µg/mL) after grafting heparin. (A) XPS wide scans and (B) evolution of the S content determined from the XPS spectra of the Cu^II^–DA/HD coatings grafted with heparin.

**Figure S6.** Anti-FXa activity of the heparin on the Cu^II^–DA/HD coatings ([DA] and [HD] constant at 1 mg/mL and 2.44 mg/mL respectively, Cu^II^–DA/HD coatings were obtained over a [CuCl_2_] range from 0 to 50 µg/mL). Data presented as mean ± SD (n=4) and analyzed using a one–way ANOVA, ^*^p < 0.05 compared to 316L SS, ^**^p < 0.01 compared to 316L SS, ^***^p < 0.001 compared to 316L SS, ^#^p < 0.05 compared to groups of 0, 12.5 and 25, ^##^p < 0.01 compared to groups of 0, 12.5 and 25, ^&^p < 0.05 compared to group 37.5 mg/l CuCl_2_.


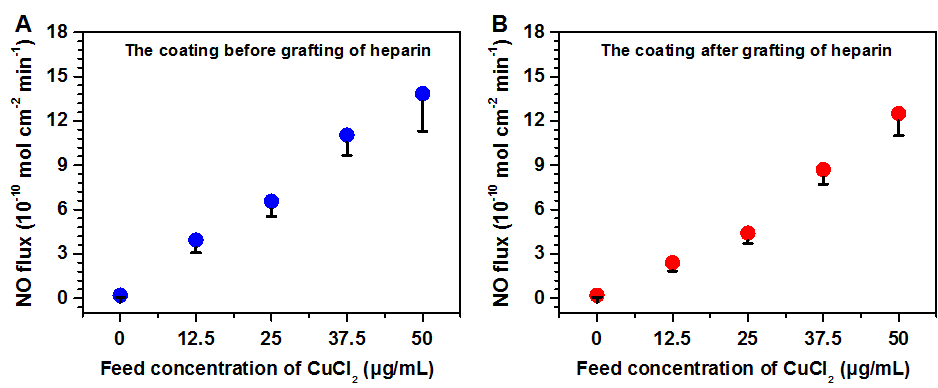


**Figure S7.** Evolution of the catalytic release of nitric oxide (NO) from the Cu^II^–DA/HD coatings without (A) and with (B) grafted heparin. Data presented as mean ± SD (n=4).


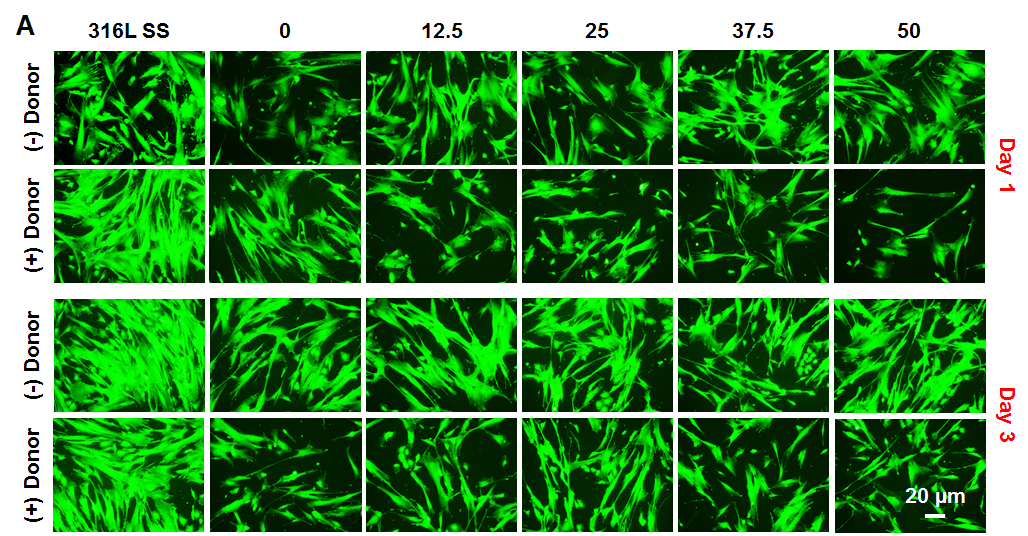

**Figure S8.** Proliferation of HUASMCs. The NO donor solution (10 μM GSNO, 10 μM GSH) was added to the cell culture medium every 4 hours for mimicking the *in vivo* blood environment. (A) Fluorescence staining of HUASMCs cultured for 1 and 3 days on 316L SS without and with Cu^II^–DA/HD coatings produced with different CuCl_2_ feed concentrations ([DA] and [HD] 1 mg/mL and 2.44 mg/mL respectively, Cu^II^–DA/HD coatings were obtained over a [CuCl_2_] range from 0 to 50 µg/mL). Proliferation of HUASMCs after 1 (B) and 3 days (C) by CCK-8 kit, respectively. Data presented as mean ± SD (n=4) and analyzed using a one–way ANOVA, ^*^p < 0.05 compared to 316L SS, ^**^p < 0.01 compared to 316L SS, ^***^p < 0.001 compared to 316L SS, ^#^p < 0.05 compared to group 0, ^##^p < 0.01 compared to group 0, ^###^p < 0.001 compared to group 0, ^&^p < 0.05 compared to group 12.5, ^&&^p < 0.01 compared to group 12.5, ^&&&^p < 0.001 compared to group 12.5.

The production of NO resulting from the addition of NO donor solution inhibited HUASMC attachment, spreading and proliferation. Note that for feed concentrations of CuCl_2_ below 25 µg/mL, the resultant Cu^II^–DA/HD coatings did not bring markedly suppress the HUASMCs proliferation especially for long time culture.

**Figure S9.** The proliferation of HUVECs. The NO donor solution (10 μM GSNO, 10 μM GSH) was added to the cell culture medium every 4 hours for mimicking the *in vivo* blood environment. (A) The fluorescence staining of HUVECs cultured for 1 and 3 days on 316L SS without and with Cu^II^–DA/HD coatings produced with different CuCl_2_ feed concentrations ([DA] and [HD] 1 mg/mL and 2.44 mg/mL respectively, Cu^II^–DA/HD coatings were obtained over a [CuCl_2_] range from 0 to 50 µg/mL). The proliferation of HUVECs after 1 (B) and 3 days (C) by CCK-8 kit, respectively. Data presented as mean ± SD (n=4) and analyzed using a one–way ANOVA, ^#^p < 0.05 compared to group 0, ^##^p < 0.01 compared to group 0, ^&^p < 0.05 compared to group 12.5, ^%^p < 0.05 compared to group 25.

The addition of NO donor solution enhanced HUVEC attachment, spreading and proliferation of the 25 and 37.5 µg/mL groups for long time culture. Note that if the initial concentrations of CuCl_2_ applied were higher than 37.5 µg/mL, the resultant Cu^II^–DA/HD coating would induce slight suppression in HUVEC proliferation.


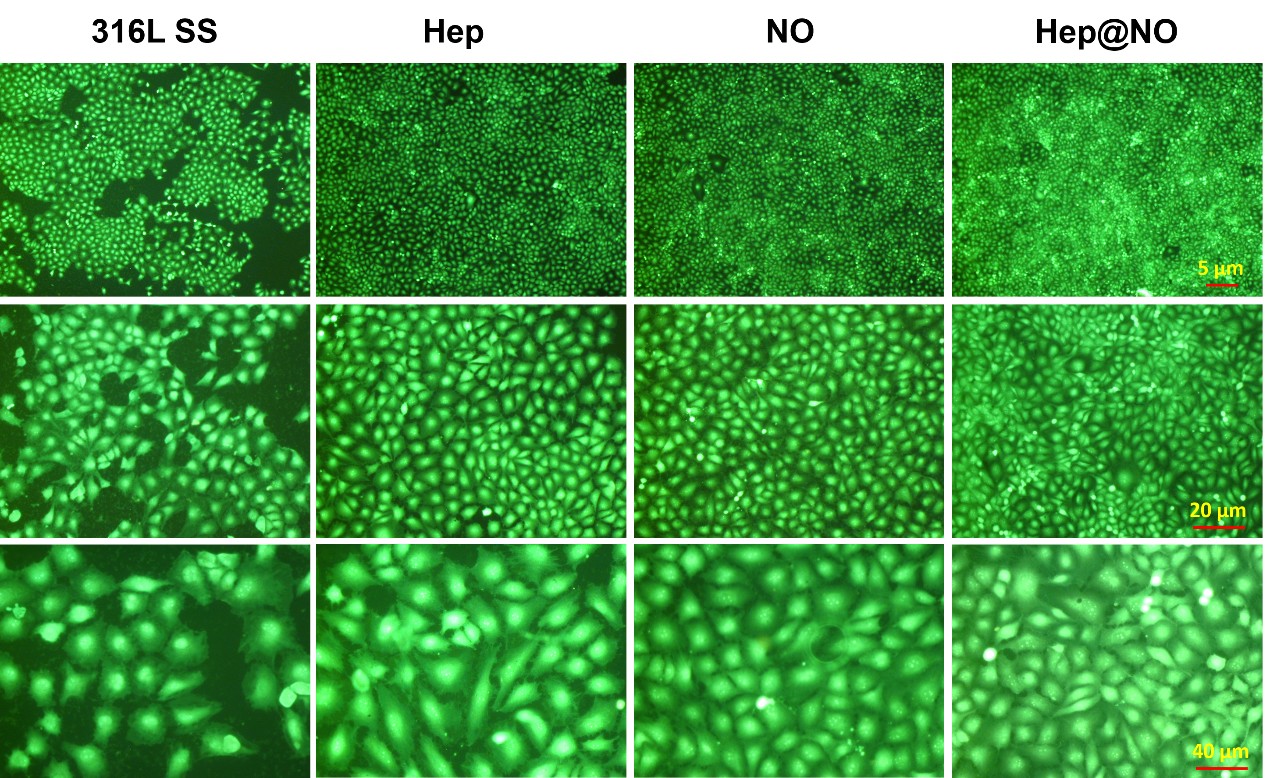


**Figure S10.** The Fluorescence staining images of HUVECs on bare 316L SS, Hep, NO and Hep@NO coatings after 3 day of culture (green: actin, blue: cell nuclei).

**Figure S11.** (A) Images of co-cultured ECs/SMCs (HUVECs (green) and HUASMCs (red)) on samples after 2 hours. The NO donor solution (10 μM GSNO, 10 μM GSH) was added to the cell culture medium for mimicking the *in vivo* blood environment. (B) The counts of ECs (Left Green) and SMCs (Right, Red) grown on the surfaces, determined from at least eight images. (C) The ratio of ECs/SMCs grown on samples surface, determined from at least eight images. Data presented as mean ± SD (n=8) and analyzed using a one–way ANOVA, *p < 0.05 and ***p < 0.001 compared to 316L SS, ^#^p < 0.05 and ^###^p < 0.001 compared to Hep, ^&^p < 0.05 and ^&&&^p < 0.001 compared to NO.

**Figure S12.** Adhesion and cGMP synthesis of platelets. The NO donor solution (10 μM GSNO, 10 μM GSH) was added to platelet rich plasma for better mimicking the *in vivo* blood environment. (A) The expression of cGMP in platelets incubated on 316L SS before and after modification by Cu^II^–DA/HD coatings ([DA] and [HD] 1 mg/mL and 2.44 mg/mL respectively, Cu^II^–DA/HD coatings were obtained over a [CuCl_2_] range from 0 to 50 µg/mL) for 30 min without (i) and with (ii) NO donor solution supplement. (B) SEM images of platelets incubated on 316L SS before and after modification by Cu^II^–DA/HD coatings ([DA] and [HD] 1 mg/mL and 2.44 mg/mL respectively, Cu^II^–DA/HD coatings were obtained over a [CuCl_2_] range from 0 to 50 µg/mL) for 30 min. Data presented as mean ± SD (n=4) and analyzed using a one–way ANOVA, ^***^p < 0.001 compared to 316L SS, ^###^p < 0.001 compared to group 0, ^&&&^p < 0.001 compared to group 12.5, ^%%%^p < 0.001 compared to group 25, ^@@@^p < 0.001 compared to group 37.5.

There were severe aggregation and activation of platelets for the Cu^II^–DA/HD coatings without NO donor solution supplement. The NO production due to NO donor solution addition resulted in significantly increased expression of cGMP of platelets, inducing significant inhibition of platelets adhesion, activation and aggregation. The amount of synthesized cGMP was a function of NO release dose, suggesting the physiological effects of NO on platelets is dose-dependent.


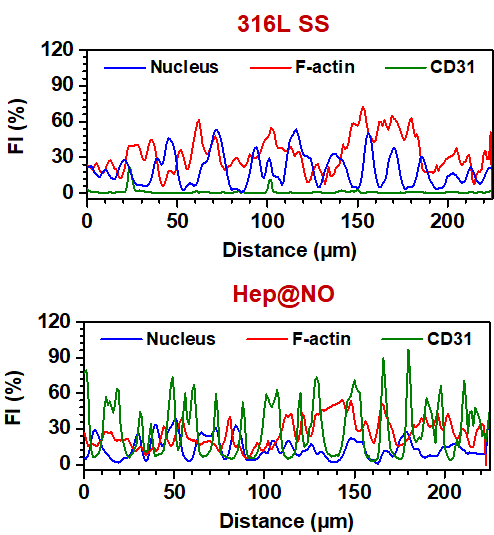


**Figure S13.** The fluorescence intensities of different components along the line segments (OA, O’B) in the images of stented-vessel shown in Figure 8A (blue: cell nucleus, red: F-actin, green: CD31).


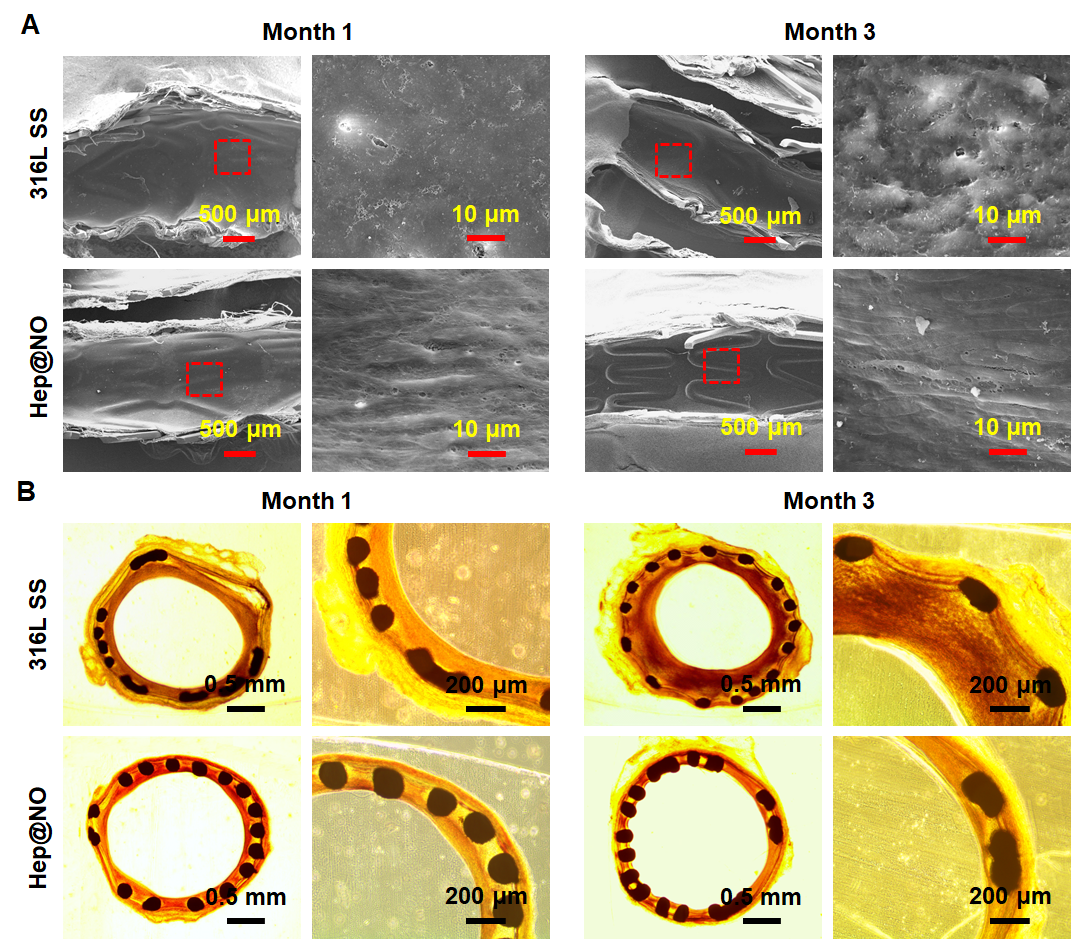


**Figure S14.**  Stent implantation *in vivo*. (A) Re-endothelialization of Hep@NO-coated and bare stents after 1 and 3 months implantation evaluated by SEM. Re-endothelialization was signiﬁcantly greater on the Hep@NO-coated stent surface than on the bare 316L SS stent surface. (B) Effect of implanted stents on ISR assessed by histomorphometric analysis.

**Table S1.** Chemical compositions of the Cu^II^-DA/HD coatings fabricated using CuCl_2_ with feed concentrations ranging from 0 to 50 μg/mL.

| CuCl_2_ (μg/mL) | C (%) | N (%) | O (%) | Cu (%) |
| --- | --- | --- | --- | --- |
| 0 | 77.0±2.9 | 9.7±1.4 | 13.4±1.2 | - |
| 12.5 | 76.3±1.7 | 9.5±0.7 | 13.3±0.9 | 0.9±0.2 |
| 25 | 75.6±2.8 | 9.9±0.5 | 13.3±1.3 | 1.2±0.2 |
| 37.5 | 75.8±1.4 | 8.8±1.1 | 13.7±1.0 | 1.7±0.2 |
| 50 | 75.4±2.6 | 9.9±0.8 | 12.8±1.4 | 1.9±0.1 |

**Table S2.** Chemical compositions of the Cu^II^-DA/HD coatings after grafting of heparin.

| CuCl_2_ (μg/mL) | C (%) | N (%) | O (%) | Cu (%) | S (%) |
| --- | --- | --- | --- | --- | --- |
| 0 | 72.8±1.4 | 9.1±0.7 | 15.9±1.2 | - | 2.2±0.2 |
| 12.5 | 70.2±1.7 | 10.4±1.0 | 16.7±1.5 | 0.7±0.1 | 2.0±0.3 |
| 25 | 71.4±2.1 | 8.3±0.6 | 17.2±2.1 | 1.2±0.3 | 1.9±0.4 |
| 37.5 | 71.9±0.9 | 8.8±1.2 | 16.0±1.3 | 1.5±0.2 | 1.8±0.2 |
| 50 | 69.2±2.6 | 10.1±0.8 | 17.3±0.9 | 1.6±0.2 | 1.8±0.5 |

**Table S3.** RNA Primers applied in this study.

| **Genes** | **Primer sequences** |
| --- | --- |
| **Human species** | |
| PECAM | Forward: 5’-CAACGAGAAAATGTCAGA-3’  Reverse: 5’-GGAGCCTTCCGTTCTAGAGT-3’ |
| VWF | Forward: 5’-CCCCTGAAGCCCCTCCTCCTA-3’  Reverse: 5’-ACGAACGCCACATCCAGAACC-3’ |
| eNOS | Forward: 5’-TCTTCCTGGACATCACCTCC-3’  Reverse: 5’-CTTCCACTCCTCGTAGCGTC-3’ |
| FGF | Forward: 5’-CTGTACTGCAAAAACGGG-3’  Reverse: 5’-AAAGTATAGCTTTCTGCC-3’ |
| GAPDH | Forward: 5′-CGGAGTCAACGGATTTGGTCGTAT-3’  Reverse: 5′-AGCCTTCTCCATGGTGGTGAAGAC-3’ |
|  | **Mouse species** |
| Tnf | Forward: 5’-CTGAACTTCGGGGTGATCGG-3’  Reverse: 5’-GGCTTGTCACTCGAATTTTGAGA-3’ |
| Il6 | Forward: 5’-ATAGTCCTTCCTACCCCAATTTCC-3’  Reverse: 5’-GATGAATTGGATGGTCTTGGTCC-3’ |
| Il18 | Forward: 5’-TGGCCGACTTCACTGTACAAC-3’  Reverse: 5’-TGGGGTTCACTGGCACTTTG-3’ |
| Il1b | Forward: 5’-TGGAGAGTGTGGATCCCAAG-3’  Reverse: 5’-GGTGCTGATGTACCAGTTGG-3’ |
| Cd86 | Forward: 5’-CTGCTCATCATTGTATGTCAC-3’  Reverse: 5’-ACTGCCTTCACTCTGCATTTG-3’ |
| Nos2 | Forward: 5’-CACCAAGCTGAACTTGAGCG-3’  Reverse: 5’-CGTGGCTTTGGGCTCCTC-3’ |
| Cd206 | Forward: 5’-AGACGAAATCCCTGCTACTG-3’  Reverse: 5’-CACCCATTCGAAGGCATTC-3’ |
| Cd163 | Forward: 5’-CGTGTGCAGTGTCCAAAAGG-3’  Reverse: 5’-CACAAACCAAGAGTGCCGTG-3’ |
| Il10 | Forward: 5’-GAGAAGCATGGCCCAGAAATC-3’  Reverse: 5’-GAGAAATCGATGACAGCGCC-3’ |
| Gapdh | Forward: 5’-TGACCACAGTCCATGCCATC-3’  Reverse: 5’-GACGGACACATTGGGGGTAG-3’ |
|  | **Rabbit species** |
| PECAM1 | Forward: 5’-GGAGTATTCGTCTACCCGTCTG-3’  Reverse: 5’-GCTCACTTCTATGGCTTGTCCT-3’ |
| eNOS | Forward: 5’-TGATGGCAAAGAGAGTGAAGG-3’  Reverse: 5’-CGAGGGACACCACATCATACT-3’ |
| VWF | Forward: 5’-TTCATGCACTGCACCTCAAGCG-3’  Reverse: 5’-GGCGTCGCACATGGGCT-3’ |
| ACTA2 | Forward: 5’-GCTGCAGCTATGTGTGAGGA-3’  Reverse: 5’-GCCATGTTCGATCGGGTACT-3’ |
| CRBP1 | Forward: 5’-CCTGGATGTCAATGTGGCCTT-3’  Reverse: 5’-CACTTGCGGTCGTCTATGCC-3’ |
| PCNA | Forward: 5’-GCCATATTGGAGATGCCGTTG-3’  Reverse: 5’-TGTAGGAGAAAGCGGAGTGGC-3’ |
| IL6 | Forward: 5’-GAAAACACCAGGGTCAGCAT-3’  Reverse: 5’-CAGCCACTGGTTTTTCTGCT-3’ |
| TNF | Forward: 5’-CGTCTCCTACCCGAACAAGG-3’  Reverse: 5’-CAGGGCAATGATCCCAAAGTA-3’ |
| IL10 | Forward: 5’-TGAGAACCACAGTCCAGCCATC-3’  Reverse: 5’-GCTCCACTGCCTTGCTCTTGT-3’ |
| GAPDH | Forward: 5’-CCGCCCAGAACATCATCCCT-3’  Reverse: 5’-GCACTGTTGAAGTCGCAGGAGA-3’ |
